# Enhanced Early Risk Stratification in Cardiogenic Shock Using Machine Learning: A Hierarchical Clustering Analysis

**DOI:** 10.1016/j.jacadv.2026.103339

**Published:** 2026-10-13

**Authors:** Shuojohn Li, Abduljabar Adi, Alejandro Quintero Villegas, Theodoros Zanos, Sami Dalati, Snehal R. Patel, Matthew Griffin, Matthew Pierce, Miguel Alvarez Villela

**Affiliations:** a North Shore University Hospital, Manhasset, New York, USA; b Northwell Cardiovascular Institute, New Hyde Park, New York, USA; c Lenox Hill Hospital, New York, New York, USA; d Feinstein Institutes for Medical Research, Manhasset, New York, USA

**Keywords:** cardiogenic shock, machine learning, phenotyping, risk stratification

## Abstract

**Background:**

Cardiogenic shock (CS) exhibits marked clinical heterogeneity, limiting the performance of conventional risk stratification tools like the Society for Cardiovascular Angiography and Interventions (SCAI) staging system. Machine learning (ML) has identified reproducible CS phenotypes that improve risk prediction, but their temporal consistency across the progression of shock remains incompletely described.

**Objectives:**

The purpose of this study was to evaluate the temporal evolution of ML-derived CS phenotypes across the first 3 intensive care unit (ICU) days and determine whether phenotype trajectories enhance mortality stratification beyond SCAI staging.

**Methods:**

In a multicenter retrospective registry of 2,906 adults with CS admitted to ICUs between 2016 and 2022, hierarchical agglomerative clustering was applied independently to clinical variables on ICU days 1, 2, and 3.

**Results:**

On day 1, 3 phenotypes emerged: Cardiac (younger, acute myocardial infarction-predominant, preserved renal function; 15% mortality), Cardio-Renal (older, non-acute myocardial infarction-predominant, isolated renal extracardiac impairment, predominantly SCAI A and B on admission; 27% mortality), and Cardio-Metabolic (severe renal and tissue malperfusion, predominantly SCAI D and E on admission; 55% mortality). Similar phenotypes persisted on day 2 and day 3. These ML-derived phenotypes enhanced mortality stratification within each SCAI stage across days 1 to 3.

**Conclusions:**

Hierarchical clustering reproduced previously identified CS phenotypes that were consistent across the first 3 days of ICU stay, providing an enhanced framework for early risk stratification. Prospective validation is required for clinical implementation.

Cardiogenic shock (CS) is a complex clinical syndrome characterized by severe impairment of cardiac function leading to reduced cardiac output and tissue hypoperfusion.^1^ A growing body of evidence shows that shock etiology, illness severity at the time of presentation, patient sex and age, as well as other characteristics, act as important risk modifiers in this syndrome.^2^, ^3^, ^4^ As a result, CS patients follow highly variable clinical trajectories, limiting the development of effective risk prediction tools and therapies.^5^ While the recently developed Cardiogenic Shock Working Group (CSWG)-Society for Cardiovascular Angiography and Interventions (SCAI) staging system has provided a valuable framework for risk stratification,^6^ SCAI classification system has shown highly variable mortality rates in different contemporary CS cohorts. It is evident that more precise risk stratification tools are needed to fully account for the variability of CS.

One emerging strategy to refine patient profiling is the use of clustering analysis, an unsupervised machine learning (ML) technique that groups heterogeneous populations into more homogeneous subgroups based on multivariate similarity. Clustering analysis identifies intrinsic patterns within data sets, determining the optimal numbers of subgroups by maximizing intercluster differences and minimizing intracluster variation.^7^ Multiple recent studies have used ML clustering in CS cohorts and have found a limited number of patient phenotypes with characteristics that seem largely reproducible.^8^, ^9^, ^10^, ^11^, ^12^

However, several limitations exist across these prior studies. Clustering models such as k-means and latent class analysis cannot natively accommodate mixed-type data^13^^,^^14^ (eg, categorical variables), constraining variable inclusion. And notably, prior phenotyping mostly selected input features based on their already known association with clinical outcomes.^8^, ^9^, ^10^ Consequently, the observed mortality gradient of these ML-derived phenotypes might largely reflect the influence of this initial feature selection process. Lastly, all previous studies have restricted phenotyping to the time of admission or have repeatedly assigned patients to the originally identified phenotypes, limiting the ability of these models to detect de novo phenotypes that can emerge over the course of the CS syndrome.

In this study, we aimed to test the reproducibility of these reported phenotypes using hierarchical clustering, a more flexible algorithm that can capture more complex clustering structures, without mortality-driven feature selection. We also aim to examine the longitudinal consistency of CS phenotypes over time by performing independent serial modeling over the first 3 days of intensive care unit (ICU) admission.

## Methods

The Northwell-Shock Registry is a multicenter retrospective observational study of patients with CS. It was approved by the Northwell Health Institutional Review Board, deemed to pose minimal risk to the study subjects, and a waiver of informed consent was obtained.

### Study population

Patients over the age of 18 admitted to 11 Northwell Health hospitals, including 4 cardiac surgery sites within our 23-hospital network, in the New York metropolitan area between January 2016 and August 2022 were screened. CS patients were defined by the presence of a principal or secondary diagnosis of CS, identified by International Classification of Diseases-10th Revision (ICD-10) code R57.0.^15^ Etiologies of CS were determined based on the primary accompanying ICD-10 diagnosis code in each patient and were based on the proposed classification by the Shock Academic Research Consortium^16^: 1) acute myocardial infarction (AMI) including ST-segment elevation myocardial infarction (STEMI) and non-STEMI; 2) heart failure (HF) associated CS including “de novo” and acute on chronic HF as well; 3) “secondary CS” due to arrhythmias, valvular, or pericardial diseases; 4) post-cardiotomy; and 5) other etiologies including mixed septic-CS and pulmonary embolism as well as other noncardiac causes.^17^ Patients with post-cardiotomy shock, mixed shock, shock due to pulmonary embolism, or other extracardiac causes were excluded from this analysis. The remaining patients were classified into AMI-CS and non-AMI-CS.^4^

### Data collection

Data were collected via an automated process through electronic health records including demographic information, comorbidities, laboratory results, and vital signs. Continuous variables were screened every 12 hours, with the worst value from each interval collected. Treatments and hospital outcomes were collected using ICD-10 procedural and diagnostic codes.^17^

### Data processing

#### Data imputation

Clustering requires complete data; imputation was therefore performed. Variables were first categorized as static: those expected to remain constant across the 3-day analytic window (eg, age, comorbidities), or dynamic: those expected to fluctuate over time (eg, vital signs, laboratory values). In our data set, static variables had minimal missingness, whereas dynamic variables showed higher missingness. For each day, patients with more than a predefined 50% missing threshold of dynamic variables were additionally excluded from imputation for that day. Missing data for the remaining patients were summarized in Supplemental Table 1 and Supplemental Figure 1.

A random forest–based imputation method with predicted mean matching (R package “missRanger”), which accommodates mixed continuous and categorical data, was used. To preserve the temporal structure across days, 3 sequential imputations were performed. First, static variables and day 1 dynamic variables were imputed across the entire cohort. Second, among patients who survived to day 2, imputed static variables and day 1 dynamic variables were used to impute day 2 dynamic variables. Third, among patients who survived to day 3, imputed static variables, day 1 and day 2 dynamic variables were used to impute day 3 dynamic variables. Imputation produced a single complete data set for each day (Supplemental Figures 2 and 3). Little's test rejected the null hypothesis of data missing completely at random (all *P* < 0.001) (Supplemental Figure 1). However, missingness was at least partially predictable from observed covariates (Supplemental Table 2), supporting the plausibility of a missing at random assumption, under which random forest imputation was performed.

Random forest–based single imputation was used because clustering analysis requires a single complete data set and a single cluster assignment per patient; pooling across multiple imputed data sets would require matching clusters between imputations, without a well-established analogue to Rubin’s rules for cluster membership. This approach is consistent with prior CS clustering studies,^8^^,^^9^ though we acknowledge single imputation does not propagate imputation uncertainty into downstream estimates.

#### Clustering analysis

After imputation, body surface area was removed from the data set due to high collinearity and clinical redundancy with body mass index (r = 0.74) (Supplemental Figure 4), all other continuous variables were tested for skewness (R package “e1071”), and those with skewness ≥1 were log-transformed. After transformation, each day's data among patients who survived to that day were submitted to Factor Analysis of Mixed Data (FAMD) (R package “FactoMineR”), which standardizes continuous variables and scales categorical levels relative to their frequency, placing both variable types in a continuous Euclidean space.

The number of retained dimensions was selected by parallel analysis using 500 simulated data sets and the 95th percentile criterion. This indicated retention of 7 dimensions for days 2 and 3. For day 1, the 8th dimension’s observed eigenvalue (1.058) did not significantly exceed the 95th percentile of simulated eigenvalue (1.058), indicating it did not meaningfully surpass chance-level variance (Supplemental Table 3, Supplemental Figure 5). To maintain consistency and comparability across clustering models, 7-dimensional FAMD coordinates were retained for all 3 days for clustering analysis. The resulting 7-dimensional coordinates explained 54.7%, 52.3%, and 51.6% of variance on days 1, 2, and 3, respectively (Supplemental Figure 5).

The retained 7-dimensional FAMD coordinates were submitted to NbClust (R package “NbClust”),^18^ using Euclidean distance and Ward.D2 linkage method to compute internal validity indices, with the optimal cluster number determined by the most frequent vote (Supplemental Table 4). Hierarchical clustering on principal components (HCPC, R package “FactoMineR”) cluster assignments were compared across 2 to 23 FAMD dimensions at each candidate k by the adjusted Rand index (R package “mclust”): the 3-cluster partition was reproducible across dimensionality, whereas the 2-cluster and 4-cluster partitions were not (Supplemental Figure 6). Final cluster assignments were generated by HCPC with k-mean consolidation on the 7-dimensional FAMD coordinates, fixed at K = 3 for all 3 days (Supplemental Figure 7). A sensitivity analysis restricted to only patients with complete preimputation data, using the identical workflow described above, yielded similar cluster characteristics (Supplemental Table 5).

### Shock stage adjudication

To assess the correlation between established shock severity stages and ML-derived phenotypes, CSWG-SCAI stages were adjudicated based on the originally published criteria using a hierarchy of variables including the imputed blood pressure, imputed lactate, imputed alanine aminotransferase (ALT), and treatment intensity.^6^

### Statistical analysis

Data analysis was conducted using R (version 4.5.3), graphs were generated using R (version 4.5.3) and GraphPad Prism (version 10.3.1). Continuous variables were reported as median and IQR, and categorical variables were presented as frequencies and percentages. Group comparisons for continuous variables were conducted using Kruskal-Wallis tests, while categorical variables were compared using chi-square tests. Random forest classifier and SHapley Additive exPlanations (SHAP) (R packages “lightgbm,” “shapviz”) method were employed to assess feature importance affecting cluster candidacies. Multivariable logistic regression models were fit for in-hospital mortality and for between-day clinical trajectories using cluster assignment, each invasive procedure, and multiplicative cluster × procedure interaction terms. These models were not further adjusted, as between-cluster differences in baseline characteristics are embedded in the cluster assignment itself, and additional covariate adjustment would be redundant.

## Results

### Phenotype characteristics

The characteristics of the overall cohort are reported in Supplemental Table 6. Three CS phenotypes were identified on day 1 of ICU admission and were named based on their clinical characteristics (with the subscript denoting day of identification). 1) Cardiac_1_ (C_1_): Including patients with the least renal dysfunction and moderate ALT and lactate elevations. This group included the youngest patients with the fewest comorbidities, the highest prevalence of men, and the highest proportion of AMI-CS with a predominance of STEMI. 2) Cardio-Renal (CR_1_): Including the oldest patients with renal dysfunction but otherwise only mildly impaired perfusion, reflected by the highest bicarbonate, along with the lowest levels of ALT and lactate. The CR_1_ group also comprises the highest proportion of non-AMI-CS. 3) Cardio-Metabolic (CM_1_): Characterized by severely impaired renal function, hepatic dysfunction, and tissue malperfusion with the highest lactate and lowest SBP. A clear gradient in mortality risk emerged between the phenotypes, with the Cardiac_1_ phenotype carrying the lowest mortality (166/1,090 [15%]), followed by CR_1_ (317/1,159 [27%]) and CM_1_ (362/657 [55%]) (Table 1).

**Table 1:** Characteristics of Day One Phenotypes

	Cardiac (n = 1,090)	Cardio-Renal (n = 1,159)	Cardio-Metabolic (n = 657)	*P* Value
Age (y)	64 [55, 73]	73 [65, 81]	71 [61, 81]	<0.001
Men (%)	769 (71)	769 (66)	382 (58)	<0.001
BMI (kg/m^2^)	26.9 [23.8, 31.0]	26.89 [23.4, 31.5]	26.17 [22.9, 30.5]	0.009
BSA (m^2^)	1.94 [1.76, 2.10]	1.92 [1.74, 2.11]	1.86 [1.69, 2.05]	<0.001
Non-AMI-CS (%)	396 (36)	1,009 (87)	330 (50)	<0.001
AMI-CS (%)	694 (64)	150 (13)	327 (50)	<0.001
STEMI (%)	430 (62)	53 (35)	199 (61)	<0.001
Death (%)	166 (15)	317 (27)	362 (55)	<0.001
Sodium (mEq/L)	137 [134, 139]	136 [132, 139]	136 [132, 140]	<0.001
Albumin (g/dL)	3.4 [3.1, 3.7]	3.2 [2.8, 3.5]	2.7 [2.3, 3.1]	<0.001
ALT (IU/L)	45 [25, 91]	28 [17, 62]	293 [64, 1,246]	<0.001
Bilirubin (mg/dL)	0.8 [0.5, 1.3]	1.0 [0.6, 1.7]	1.3 [0.8, 2.3]	<0.001
Creatinine (mg/dL)	1.17 [0.91, 1.54]	2.07 [1.39, 3.12]	2.28 [1.56, 3.29]	<0.001
eGFR (mL/min/1.73m^2^)	65 [46, 85]	31 [18, 50]	26 [18, 42]	<0.001
Hemoglobin (g/dL)	12.1 [10.1, 13.5]	10.1 [8.8, 11.7]	9.7 [8.2, 11.6]	<0.001
WBC (k/uL)	14.4 [10.9, 19.1]	9.9 [7.4, 13.1]	18.4 [13.5, 24.2]	<0.001
Platelet (k/uL)	193 [147, 247]	166 [123, 217]	142 [95, 201]	<0.001
Lactate (mmol/L)	2.50 [1.6, 4.2]	2.1 [1.4, 3.4]	8.9 [5.6, 13.2]	<0.001
Bicarbonate (mEq/L)	20 [18, 22]	21 [19, 24]	15 [12, 17]	<0.001
HR (beats/min)	88 [76, 101]	89 [75, 105]	92 [78, 109]	<0.001
SBP (mm Hg)	96 [85, 109]	88 [76, 101]	80 [63, 94]	<0.001
DBP (mm Hg)	53 [46, 61]	48 [40, 55]	42 [31, 52]	<0.001
CO (L/min)	4.0 [3.3, 5.0]	4.2 [3.2, 5.3]	4.1 [3.0, 5.3]	0.322
Cardiac index (L/min/m^2^)	2.1 [1.7, 2.6]	2.2 [1.7, 2.6]	2.1 [1.7, 2.8]	0.754
CVP (mm Hg)	11 [7, 16]	10 [7, 15]	12 [8, 17]	0.037
PAS (mm Hg)	37 [30, 46]	40 [32, 53]	37 [31, 45]	0.002
PAD (mm Hg)	18 [14, 23]	19 [14, 24]	18 [14, 23]	0.152
HTN (%)	620 (57)	946 (82)	454 (69)	<0.001
CKD (%)	64 (6)	678 (59)	173 (26)	<0.001
HF (%)	485 (45)	1,046 (90)	331 (50)	<0.001
CAD (%)	370 (34)	683 (59)	256 (39)	<0.001
DM (%)	342 (31)	666 (57)	270 (41)	<0.001
AF (%)	211 (19)	764 (66)	204 (31)	<0.001
CCI	4 [3, 6]	7 [5, 9]	6 [4, 8]	<0.001

AF = atrial fibrillation; ALT = alanine transaminase; AMI-CS = acute myocardial infarction–related cardiogenic shock; BMI = body mass index; BSA = body surface area; CAD = coronary artery disease; CCI = Charlson Comorbidity Index; CKD = chronic kidney disease; CO = cardiac output; CVP = central venous pressure; DBP = diastolic blood pressure; DM = diabetes mellitus; eGFR = estimated glomerular filtration rate; HF = heart failure; HR = heart rate; HTN = hypertension; PAD = pulmonary artery diastolic pressure; PAS = pulmonary artery systolic pressure; SBP = systolic blood pressure; STEMI = ST-segment elevation myocardial infarction; WBC = white blood cell.

Random forest classifiers and SHAP analysis were used to assess feature importance in determining cluster membership. The top features predicting the Cardiac_1_ phenotype were 1) lower serum creatinine; 2) absence of pre-existing chronic kidney disease, atrial fibrillation, or HF; and 3) AMI etiology of CS. In contrast, for CR_1_ phenotype, these were: 1) presence of pre-existing chronic kidney disease, atrial fibrillation, or HF; 2) non-AMI etiology of CS; 3) lower white blood cell counts; 4) lower serum lactate and higher bicarbonate, and for the CM_1_ phenotype, these were: 1) higher lactate; 2) lower serum bicarbonate; and 3) higher ALT (Supplemental Figure 8).

### Longitudinal phenotyping

On day 2 and 3 of ICU admission, 3 phenotypes were identified with defining clinical characteristics that were similar to those observed on day 1, and they were again labeled (with the subscript denoting day of identification) C_2_, CR_2_, CM_2_, C_3_, CR_3_, and CM_3_, respectively (Supplemental Table 7). A random forest classifier and SHAP analysis used to assess feature importance of determining cluster membership on day 2 and 3 and revealed mostly similar feature importance trends as on day 1 (Supplemental Figure 8).

### Invasive therapies

Invasive therapies utilization varied significantly across phenotypes. The C_1_ phenotype had the highest overall use of mechanical circulatory support (MCS, 576/1,090 [53%] vs CR_1_ 292/1,159 [25%] vs CM_1_ 251/657 [38%]), primarily intra-aortic balloon pump (IABP, 462/1,090 [42%] vs CR_1_ 222/1,159 [19%] vs CM_1_ 158/657 [24%]). Conversely, the CM_1_ phenotype had the highest rates of renal replacement therapy (101/657 [25%] vs CR_1_ 256/1,159 [22%] vs C_1_ 57/1,090 [5%]) and mechanical ventilation (447/657 [68%] vs CR_1_ 364/1,159 [31%] vs C_1_ 418/1,090 [38%]) (Figure 1). Similar trends were noted across all 3 days of analysis (Supplemental Figure 9).

**Figure 1:**
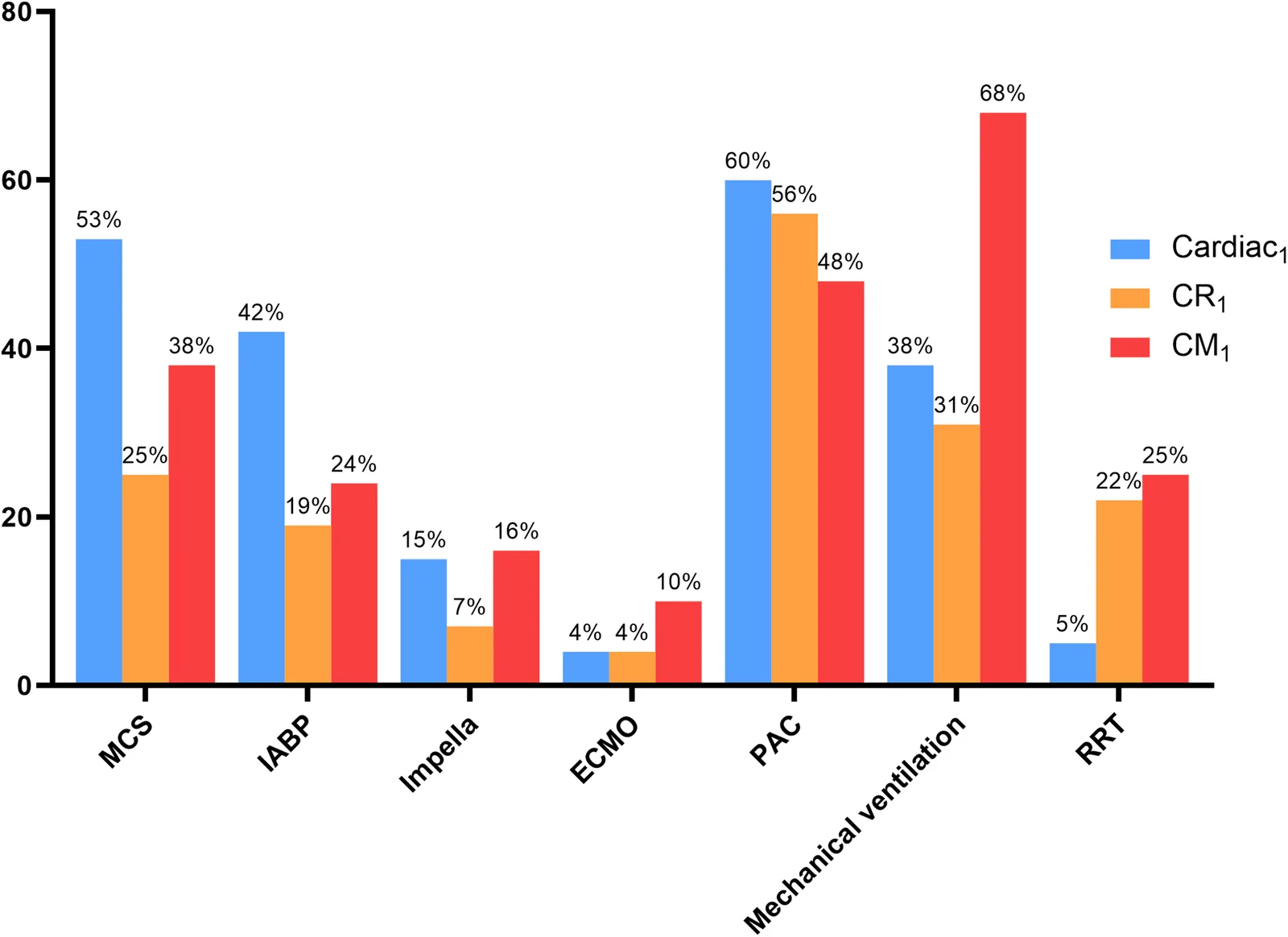
Invasive Therapy Use by Phenotype Bar charts showing utilization of mechanical circulatory support and pulmonary artery catheterization across day 1 phenotypes; cardiac phenotype patients on day 1 had the most mechanical circulatory support use, predominantly intra-aortic balloon pump. *P* value <0.05 for all chi-square tests. CM = cardio-metabolic; CR = cardio-renal; ECMO = extracorporeal membrane oxygenation; IABP = intra-aortic balloon pump; MCS = mechanical circulatory support; PAC = pulmonary artery catheter; RRT = renal replacement therapy.

Among day 1 phenotypes, Impella was associated with higher mortality in the CM_1_ phenotype (OR: 2.00; 95% CI: 1.23-3.23), *P* = 0.005), whereas pulmonary artery catheter (PAC) was associated with lower mortality among the CR_1_ phenotype (OR: 0.73; 95% CI: 0.55-0.97, *P* = 0.031). However, no significant phenotype-based modification was detected after adjusting for multiple comparisons (Figure 2).

**Figure 2:**
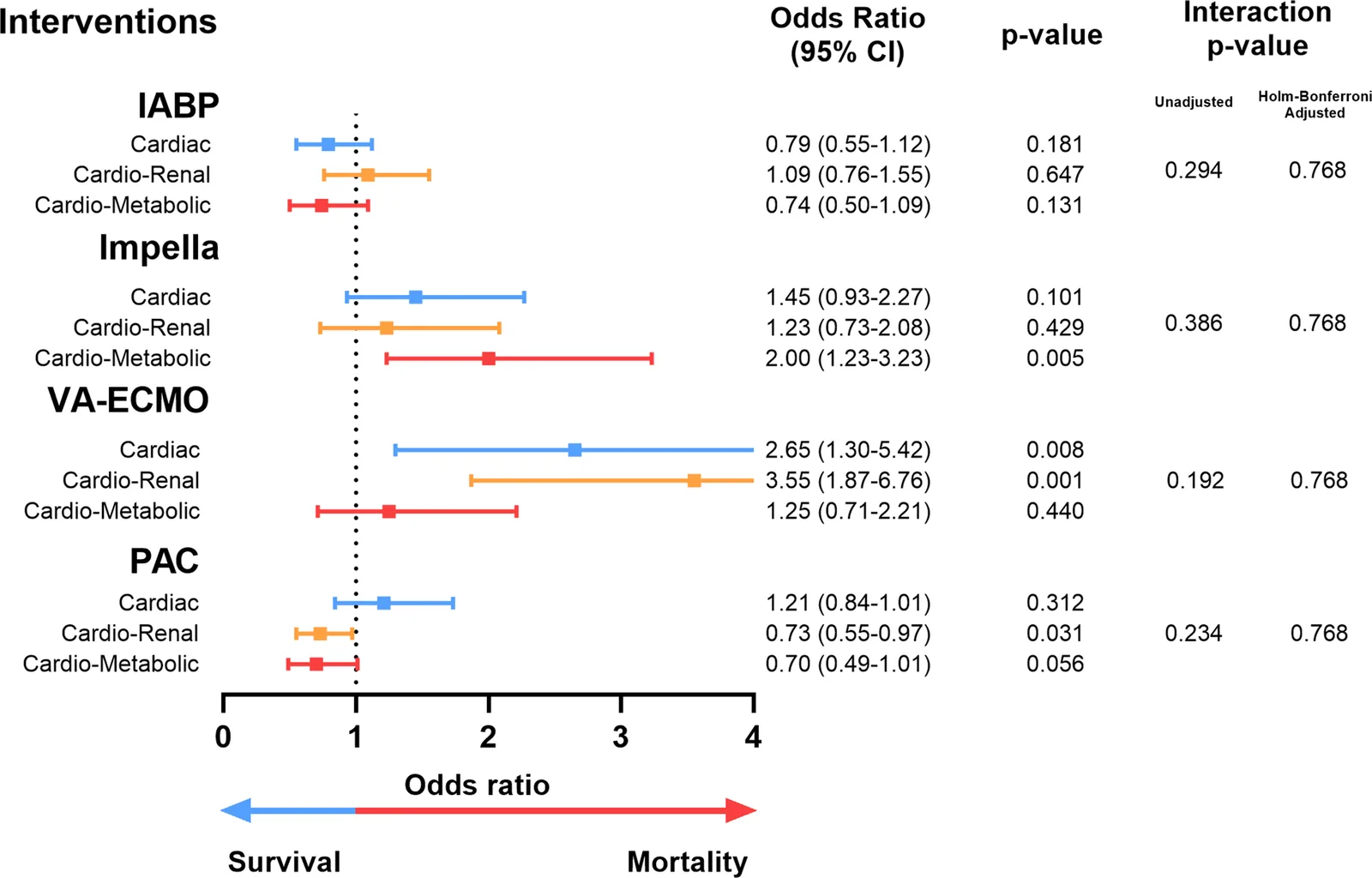
Phenotype-Specific Associations of Invasive Therapies With Mortality Forest plot of ORs for in-hospital mortality associated with each invasive therapy, estimated from logistic regression with phenotype × therapy interaction terms and stratified by day 1 phenotype. Veno-arterial extracorporeal membrane oxygenation was associated with higher mortality among Cardiac and Cardio-Renal patients but not among Cardio-Metabolic patients, Impella was associated with higher mortality among Cardio-Metabolic patients, and pulmonary artery catheter was associated with lower mortality among Cardio-Renal patients. No interaction term reached statistical significance after Holm-Bonferroni correction. VA-ECMO = veno-arterial extracorporeal membrane oxygenation; other abbreviations as in Figure 1.

### Phenotype progression

The identified 3-cluster structure was consistent over time, and the majority of patients in each cluster received the same cluster assignment at days 1 to 2 and days 2 to 3 (Figure 3).

**Figure 3:**
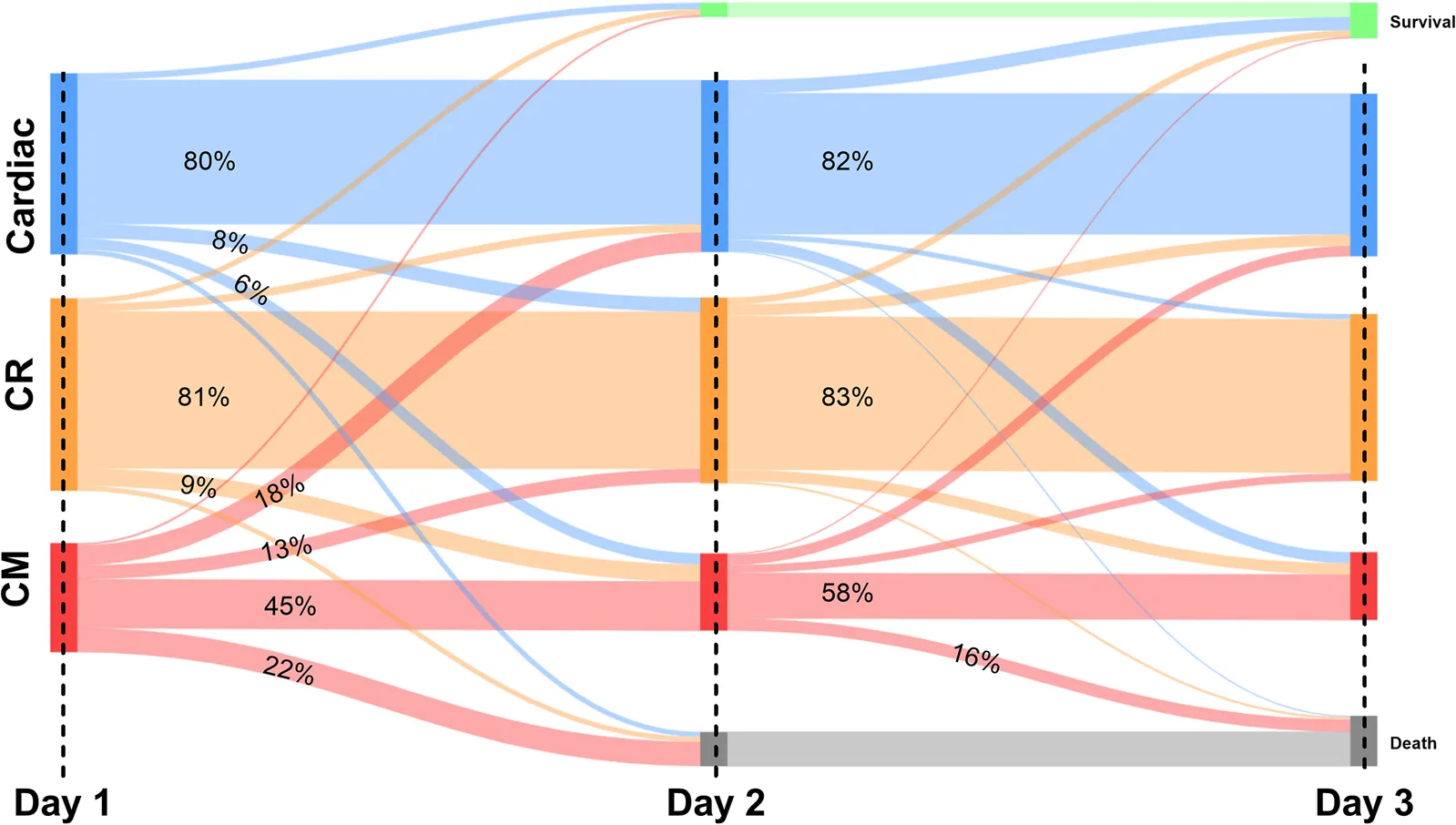
Phenotype Progression Over First Three Intensive Care Unit Days Sankey diagram depicting day-to-day phenotypes assignment during the first 3 days of intensive care unit admission. Abbreviations as in Figure 1.

Between-day worsening clinical trajectories were defined as moving from a low-mortality phenotype to a higher-mortality phenotype or in-hospital death, while lack of worsening trajectories was defined as stable clinical trajectories between days. From day 1 to 2, the use of IABP (OR: 0.23; 95% CI: 0.11-0.49; *P* < 0.001), veno-arterial extracorporeal membrane oxygenation (OR: 0.16; 95% CI: 0.03-0.70; *P* = 0.015), and PAC (OR: 0.21; 95% CI: 0.08-0.54; *P* = 0.001) are associated with a stable clinical trajectory among CM_1_ (defined as migrating to C_2_, CR_2_, or remain in CM_2_), there are significant phenotype-based effect modifications for these devices (interaction *P* = 0.007, 0.005, 0.002 for IABP, veno-arterial extracorporeal membrane oxygenation, and PAC, respectively). Whereas the use of PAC was associated with stable clinical trajectory among CM_2_ (OR: 0.53; 95% CI: 0.29-0.95; *P* = 0.034, interaction *P* = 0.04), and use of Impella was associated with worsening clinical trajectory among CM_2_ (OR: 2.86; 95% CI: 1.19-6.87; *P* = 0.019). Although the interaction *P* value for Impella did not reach statistical significance after Holm-Bonferroni adjustment (Figure 4).

**Figure 4:**
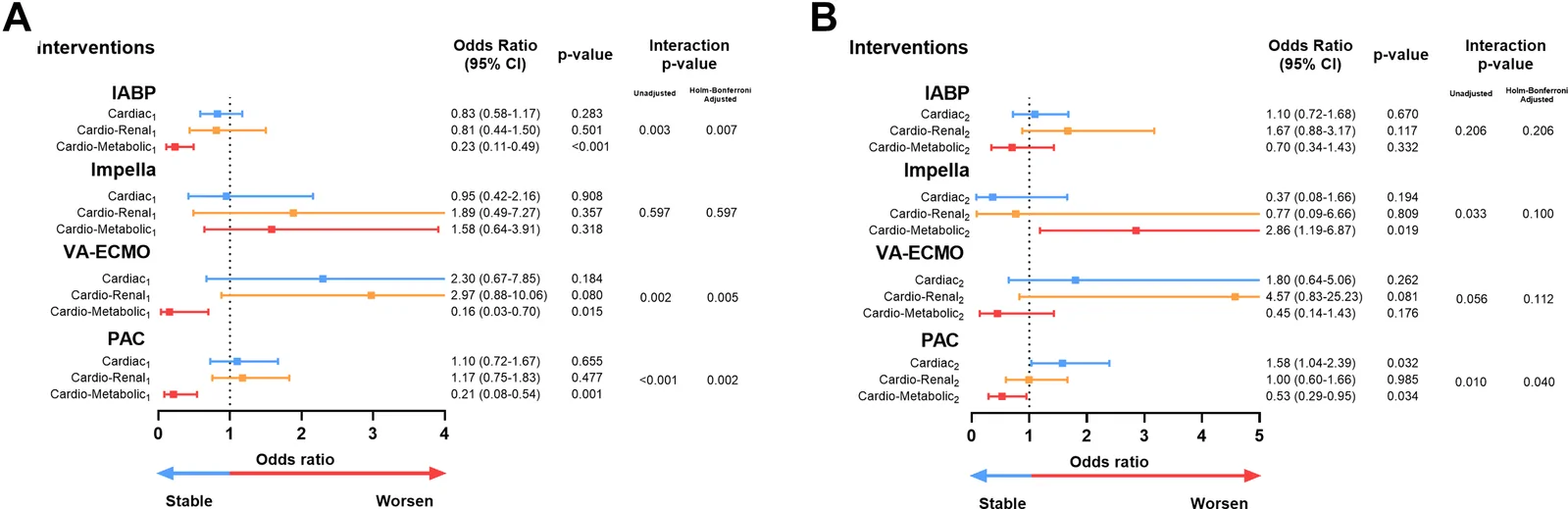
Therapy Impact on Phenotype Transitions Logistic regression with phenotype × therapy interaction terms assessing the association of invasive therapies with phenotypic transition between days, stratified by the phenotype at the start of each interval. Worsening clinical trajectory is defined as migration from a lower mortality phenotype to a higher mortality one or hospital mortality. Stable clinical trajectory is defined as migration from a higher mortality phenotype to a lower mortality one or survived to discharge. Between day 1 to 2, the use of IABP, VA-ECMO, and PAC was associated with stable trajectory among CM1 patients (A), between day 2 to 3 the use of Impella was associated with worsening trajectory among CM2 patients (B). Abbreviations as in Figures 1 and 2.

### Phenotypes and shock stages

CSWG-SCAI shock stages were adjudicated at hospital admission, during the first 3 days of ICU stay, and at the time of maximum disease severity. Notably, the distribution of SCAI stages in each phenotype varied significantly at the time of hospital and ICU admission. The CR_1_ group had the highest proportion of patients in stages A and B, while CM_1_ had the highest proportion of patients in stages D and E, and the C_1_ group had a more even distribution of stages. The distribution of maximal SCAI stages was also significantly different with the proportion of stage E patients increasing progressively from C_1_ to CR_1_ to CM_1_, reflecting the stepwise increase in mortality rate (Figure 5).

**Figure 5:**
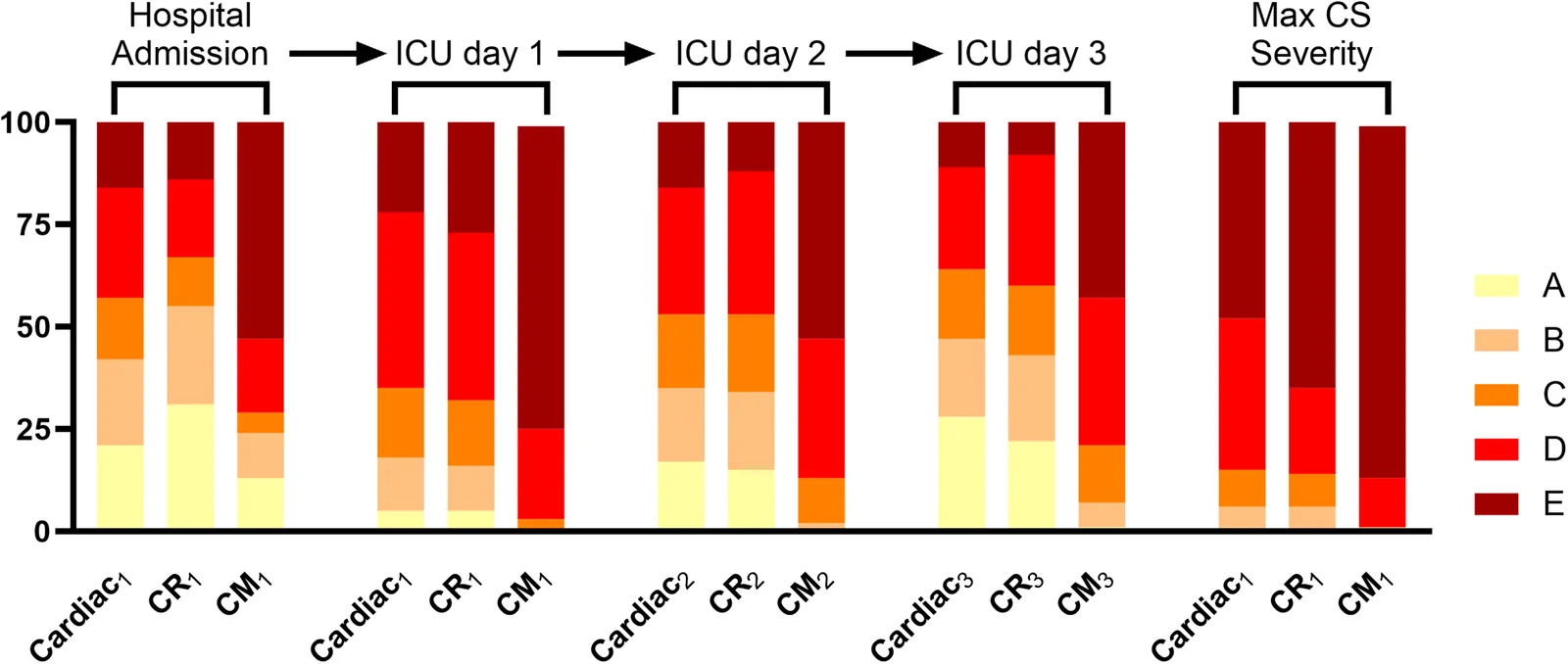
Society for Cardiovascular Angiography and Interventions Shock Stage Distribution by Day One Phenotype Stacked bar charts showing the distribution of Cardiogenic Shock Working Group-Society for Cardiovascular Angiography and Interventions shock stages at hospital admission, day 1 to 3 of intensive care unit admission and at maximum disease severity, stratified by phenotype of each day. CS = cardiogenic shock; ICU = intensive care unit; other abbreviations as in Figure 1.

Like in prior one study by Zweck et al,^8^ when combined with CSWG-SCAI shock stages, ML-derived phenotypes offered enhanced day-to-day mortality risk stratification throughout the first 3 days of ICU stay. Among patients with the same SCAI shock stage, those classified as the CM phenotype on each day experienced the highest mortality, while those classified to the Cardiac phenotype had the lowest mortality. The highest mortality rates were consistently observed in SCAI stage E patients with the CM phenotype, reaching a highest of 67% (168/249) on day 2. Meanwhile, the lowest mortality rate was observed in SCAI C patients with the cardiac phenotype reaching lowest of 12% across all 3 days (22/182, 22/186, 20/167, for days 1, 2, and 3, respectively) (Figure 6).

**Figure 6:**
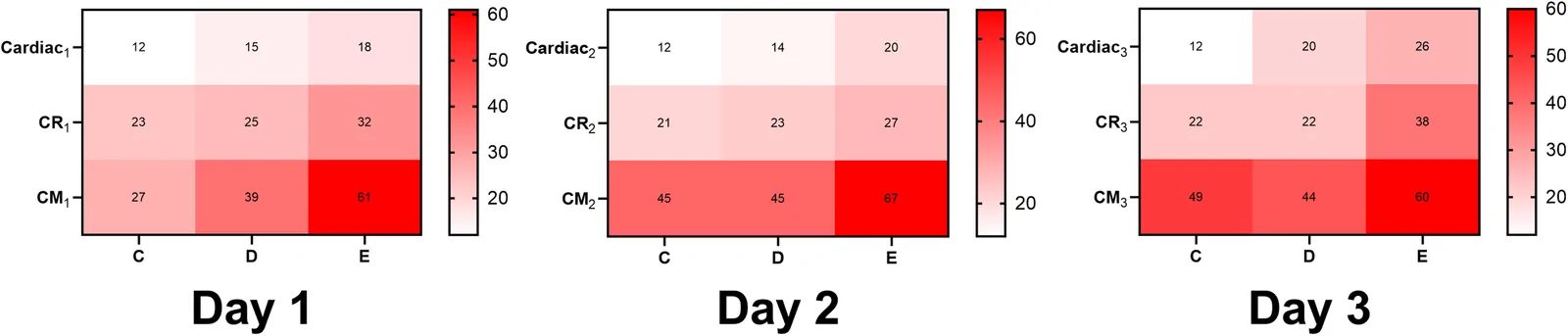
Mortality by Society for Cardiovascular Angiography and Interventions Stage and Phenotype Across Three Days In-hospital mortality stratified by Cardiogenic Shock Working Group-Society for Cardiovascular Angiography and Interventions shock stage and phenotypes on days 1, 2, and 3 of intensive care unit admission, demonstrating that machine learning phenotypes enhance mortality risk stratification within each Society for Cardiovascular Angiography and Interventions stage. Abbreviations as in Figure 1.

## Discussion

This large multicenter study used hierarchical clustering, a type of unsupervised ML, to derive serial patient phenotypes in CS. Our main findings were: 1) at the time of ICU admission, 3 phenotypes were identified; 2) the identified phenotypes largely correspond to those described in prior studies that used different ML techniques and input variables selection,^8^^,^^11^, ^12^, ^13^ indicating the robust reproducibility of these patient profiles; 3) unrestricted de novo longitudinal clustering revealed a relative consistency of cluster clinical characteristics during the first 3 days of ICU admission, with the majority of patients retaining their day 1 phenotype assignment; 4) patient assignment to these phenotypes enhanced the mortality risk stratification provided by the CSWG-SCAI staging system at each time point; 5) discrete differences in treatment strategies were seen across phenotypes, suggesting the implicit recognition of these patient profiles by clinicians (Central Illustration).

**Central Illustration:**
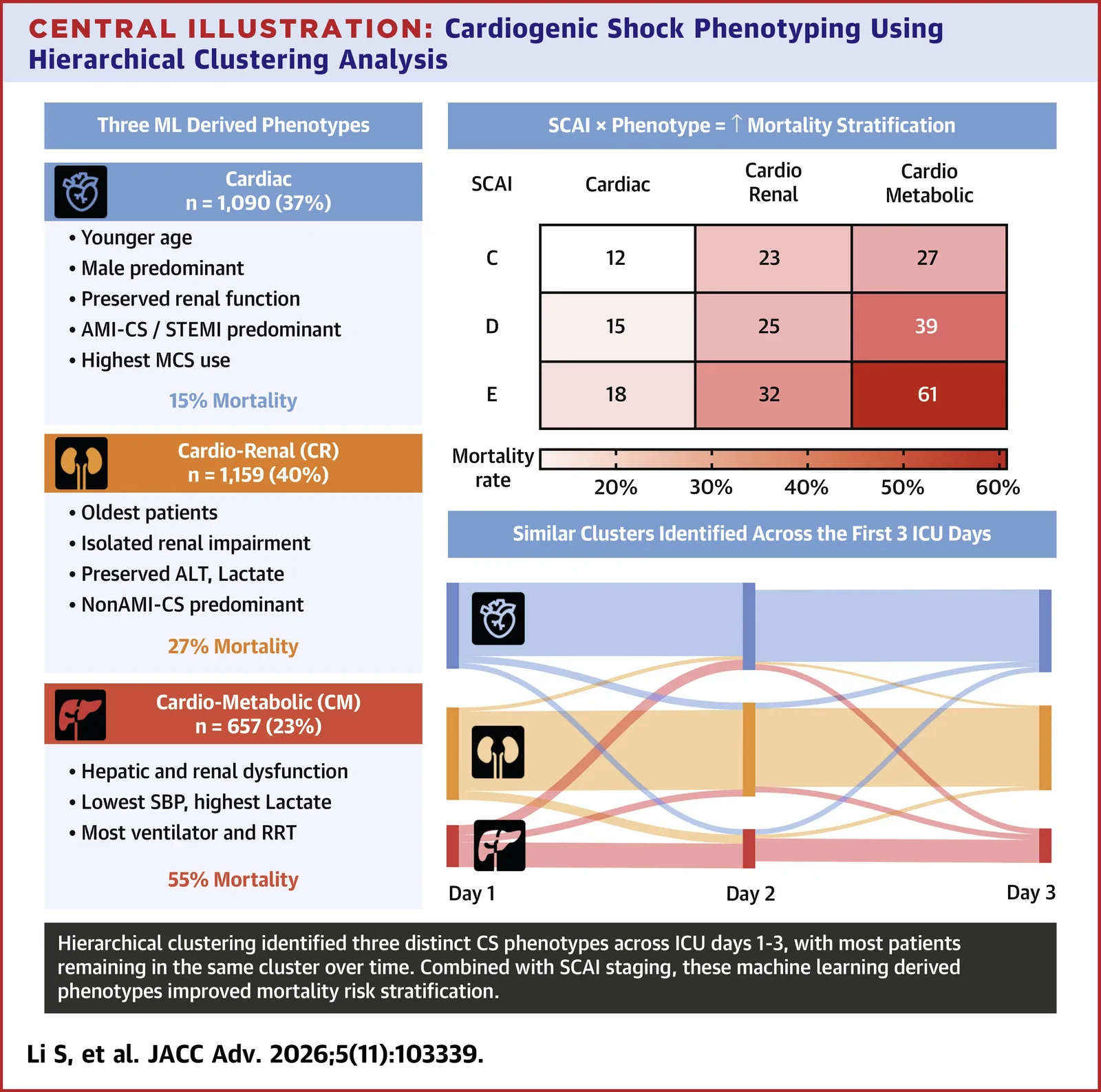
Cardiogenic Shock Phenotyping Using Hierarchical Clustering Analysis Hierarchical clustering identified three distinct cardiogenic shock phenotypes across intensive care unit days 1 to 3, with most patients remaining in the same phenotype over time. Combined with Society for Cardiovascular Angiography and Interventions staging, these machine learning–derived phenotypes improved mortality risk stratification. ALT = alanine aminotransferase; AMI = acute myocardial infarction; SBP = systolic blood pressure; SCAI = Society for Cardiovascular Angiography and Interventions; ML = machine learning; STEMI = ST-segment elevation myocardial infarction; other abbreviations as in Figures 1 and 5.

Our study adds to the growing evidence of the potential utility of ML to enhance patient phenotyping in CS.^19^ Our approach was novel in the use of hierarchal clustering, as well as independent clustering applied each day during the early ICU admission period to identify the progression of the initial phenotypes. Hierarchical clustering is an unsupervised ML method that builds a tree-like structure (dendrogram) by iteratively merging similar observations into subgroups based on their multivariate distances (Supplemental Figure 10). Importantly, prior studies^8^^,^^11^, ^12^, ^13^, ^14^ employed a feature preselection scheme based on associations with mortality derived from regression modeling, making interpreting the ML-derived phenotypes’ association to mortality challenging, as the risk gradient observed might be related to the feature selection process itself. To this end, we performed dimensional reduction using FAMD, allowing all variables to contribute to the final clustering model to a varying degree. Despite using a different clustering and dimension reduction method, the phenotypes identified in these studies closely resemble those seen in our analysis, and importantly, preserved the mortality gradient observed in prior studies. This consistency underscores the potential of ML clustering to enhance prospective risk stratification for this highly heterogeneous condition and inform future clinical trial design.

However, a key limitation of prior studies has been the reliance on a single phenotyping analysis conducted at the time of admission.^8^^,^^11^, ^12^, ^13^, ^14^ Although one study attempted to longitudinally reassign patients within the phenotypes identified at admission over the early course of their hospitalization,^20^ this approach relies on a fixed phenotypes generated at the time of admission and cannot determine whether these phenotypes represent stable clusters, or rather dynamic groups that merge or separate over time. To this end, our study performed de novo unrestricted clustering on day 2 and day 3 of ICU admission. This analysis revealed a consistent 3-cluster structure throughout the first 3 days of ICU stay, with clusters of similar clinical characteristics and patient composition. These findings carry important clinical implications, suggesting that these phenotypes may be static patient profiles that can be reliably determined at ICU admission to predict downstream patient outcomes. However, a key limitation to this study is the short analysis window, limited only to the first 3 days of ICU stay. It remains unexplored whether the observed 3 phenotypes can merge or separate over time later in the course.

Hierarchical clustering also resulted in early improved discrimination of mortality risk compared to the SCAI-CSWG stages. For instance, CR_1_ phenotype had the highest proportion of stages A or B shock during early hospital course based on the traditional paradigm of hypotension and hypoperfusion used by the staging system. Moreover, CR had similar SCAI composition compared to Cardiac phenotype throughout the analytic window. Despite this similarity in SCAI staging, CR clearly had a higher mortality risk compared to Cardiac phenotype. This discrepancy suggests these ML-derived phenotypes might capture residual mortality risk that is missed by the traditional SCAI staging.

Another key unanswered question is whether specific phenotypes derive differential benefits from specific therapies. One previous study examined phenotype-treatment interaction but was limited by unmeasured confounding.^21^ In our cohort, we observed phenotype-based differential impact of mortality and their potential impact on phenotype transitions of each device. Although these observed differential impacts may suggest phenotype-specific differential benefits from therapies, they could also arise from residual confounding related to patient characteristics, shock severity, or clinician-driven selection of therapies. Therefore, these findings should be interpreted as hypothesis-generating and warrant further investigation of phenotype-based therapies. Nonetheless, the distinct therapy utilization patterns across phenotypes imply a degree of tacit recognition of these phenotypes in clinical practice, with bias toward certain therapies for certain patient profiles.

Many other questions remain to be answered before these ML phenotyping models can be adopted in clinical practice. Although existing ML models are already capable of real-time phenotyping, none of the existing models were tested prospectively and thus remain hypothesis generating. Prospective validation studies are needed to assess whether real-time phenotyping can reliably predict mortality risk and to identify all the practical barriers to clinical deployment of these tools.^19^

### Study Limitations

First, the retrospective observational design precludes causal inference and limits the exploration of real-world applicability. Second, invasive hemodynamics were not used for imputation and clustering analysis due to a significant proportion of patients without PAC monitoring. Additionally, reliance on ICD-10 codes may introduce misclassification bias, although these codes have been validated in prior studies.^22^^,^^23^ Third, while the vast majority of patients were treated in centers with MCS capability within the same health system, site and operator heterogeneity as well as interhospital transfer were not accounted for in our clustering models.

## Conclusions

Unsupervised hierarchical clustering identified CS phenotypes that closely mirrored those from prior studies employing alternative clustering methods and variable sets. As a key novel contribution, de novo longitudinal clustering revealed these phenotypes remained relatively static over time during the first 3 ICU days. These ML-derived phenotypes significantly enhanced hospital mortality risk prediction beyond the established CSWG-SCAI staging system. Given their consistent reproducibility across CS cohorts and superior prognostic performance, prospective validation studies are now essential to enable clinical adoption or integration into trial design.

Perspectives **COMPETENCY IN PATIENT CARE:** Cardiogenic shock exhibits highly heterogeneous clinical trajectories, making the development of uniform management strategies challenging. Clinicians should be aware of the rapidly evolving role of ML in facilitating early risk stratification and phenotype-guided decision-making in cardiogenic shock.

**TRANSLATIONAL OUTLOOK:** Prospective studies are needed to evaluate the efficacy of ML-derived phenotyping for early risk stratification in cardiogenic shock. Additionally, characterizing the phenotype-specific effects of MCS may inform future trial design and improve patient selection for device-based therapies.

## Funding support and author disclosures

The authors have reported that they have no relationships relevant to the contents of this paper to disclose.
