# Supplementary material for: Enhanced Early Risk Stratification in Cardiogenic Shock Using Machine Learning: A Hierarchical Clustering Analysis
Source: JACC Adv. 2026 Oct 13;5(11):103339. doi: 10.1016/j.jacadv.2026.103339 (PMC13685971; doi:10.1016/j.jacadv.2026.103339)
Supplement: Supplementary file 1 [file mmc1.docx]

**Supplemental Material**

| **Table S1. Imputation input features and missingness** | | | | |
| --- | --- | --- | --- | --- |
| **Variables** | | **Pre-imputation missingness (%)**  **(n = 2096)** | | |
| **Static** | Age | 0 (0) | | |
|  | BMI | 76 (3) | | |
|  | BSA* | 72 (2) | | |
|  | Sex | 0 (0) | | |
|  | CS-etiology | 0 (0) | | |
|  | HTN | 0 (0) | | |
|  | CKD | 0 (0) | | |
|  | HF | 0 (0) | | |
|  | CAD | 0 (0) | | |
|  | DM | 0 (0) | | |
|  | AF | 0 (0) | | |
|  | | **Day 1**  **(n = 2906)** | **Day 2**  **(n = 2619)** | **Day 3**  **(n = 2392)** |
| **Dynamic** | Lactate | 402 (13.8) | 796 (30.4) | 959 (40.1) |
|  | Creatinine | 0 (0) | 11 (0.4) | 12 (0.5) |
|  | ALT | 107 (3.7) | 408 (15.6) | 488 (20.4) |
|  | Sodium | 0 (0) | 9 (0.3) | 10 (0.4) |
|  | Bilirubin | 106 (3.7)) | 402 (15.3) | 476 (19.9) |
|  | Bicarbonate | 5 (0.2) | 14 (0.5) | 10 (0.4) |
|  | Albumin | 96 (3.3) | 394 (15.0) | 467 (19.5) |
|  | Hemoglobin | 4 (0.1) | 21 (0.8) | 16 (0.7) |
|  | WBC | 4 (0.1) | 22 (0.8) | 15 (0.6) |
|  | Platelet | 10 (0.3) | 27 (1.0) | 21 (0.9) |
|  | Heart rate | 15 (0.5) | 8 (0.3) | 10 (0.4) |
|  | SBP | 71 (2.4) | 90 (3.4) | 72 (3.0) |
|  | DBP | 72 (2.5) | 91 (3.5) | 73 (3.1) |
| AF = atrial fibrillation; BSA = body surface area; CAD = coronary artery disease; CKD = chronic kidney disease; CS = cardiogenic shock; DBP = diastolic blood pressure; DM = diabetes mellitus; eGFR = estimated glomerular filtration rate; HF = heart failure; HR = heart rate; HTN = hypertension; SBP = systolic blood pressure; STEMI = ST-segment elevation myocardial infarction; WBC = white blood cell.  *After imputation, BSA was removed from downstream analysis due to high collinearity with BMI | | | | |

| **Table S2. Data missingness predictability by observed variables** | | | | | | | | | | | | | | | | | |
| --- | --- | --- | --- | --- | --- | --- | --- | --- | --- | --- | --- | --- | --- | --- | --- | --- | --- |
| **Variable** | **Predictor** | **OR** | **Low CI** | **High CI** | **p value** | **Variable** | **Predictor** | **OR** | **Low CI** | **High CI** | **p value** | **Variable** | **Predictor** | **OR** | **Low CI** | **High CI** | **p value** |
| **lactate1** | age | 1.1590912 | 0.9974228 | 1.3496112 | 5.41E-02 | **lactate2** | age | 1.3775288 | 1.2302778 | 1.5447066 | 2.08E-08 | **lactate3** | age | 1.386028 | 1.2407695 | 1.5506008 | 5.39E-09 |
| **lactate1** | sexMale | 0.8430747 | 0.6302883 | 1.1310369 | 2.54E-01 | **lactate2** | sexMale | 0.7000463 | 0.5637 | 0.8693293 | 1.26E-03 | **lactate3** | sexMale | 0.6194939 | 0.5003819 | 0.7664533 | 1.03E-05 |
| **lactate1** | bmi | 1.0120943 | 0.8879964 | 1.1497148 | 8.55E-01 | **lactate2** | bmi | 1.0780573 | 0.9757898 | 1.1911954 | 1.39E-01 | **lactate3** | bmi | 1.0963882 | 0.9928391 | 1.2113326 | 6.91E-02 |
| **lactate1** | csetiologyami | 1.9216848 | 1.4065972 | 2.6323868 | 3.91E-05 | **lactate2** | csetiologyami | 1.0832235 | 0.8649695 | 1.3561571 | 4.86E-01 | **lactate3** | csetiologyami | 1.0411599 | 0.8349665 | 1.298184 | 7.20E-01 |
| **lactate1** | htn1 | 0.8498076 | 0.6391057 | 1.1348723 | 2.68E-01 | **lactate2** | htn1 | 1.0127462 | 0.813009 | 1.2632672 | 9.10E-01 | **lactate3** | htn1 | 1.028177 | 0.8274495 | 1.2784181 | 8.02E-01 |
| **lactate1** | ckd1 | 1.1646388 | 0.8404905 | 1.6090001 | 3.58E-01 | **lactate2** | ckd1 | 0.9392445 | 0.7409876 | 1.189427 | 6.03E-01 | **lactate3** | ckd1 | 0.821459 | 0.6512812 | 1.0351798 | 9.56E-02 |
| **lactate1** | hf1 | 0.5450051 | 0.4022106 | 0.7375957 | 8.47E-05 | **lactate2** | hf1 | 0.571619 | 0.4535848 | 0.7193988 | 1.78E-06 | **lactate3** | hf1 | 0.68466 | 0.5456708 | 0.8582122 | 1.01E-03 |
| **lactate1** | cad1 | 0.8250047 | 0.6297957 | 1.0790541 | 1.60E-01 | **lactate2** | cad1 | 1.1217415 | 0.9155917 | 1.3747773 | 2.68E-01 | **lactate3** | cad1 | 0.9143567 | 0.7478998 | 1.1178874 | 3.82E-01 |
| **lactate1** | dm1 | 1.1447036 | 0.8707892 | 1.5046977 | 3.33E-01 | **lactate2** | dm1 | 0.8848435 | 0.7210565 | 1.08536 | 2.41E-01 | **lactate3** | dm1 | 0.8157616 | 0.6677793 | 0.9961362 | 4.57E-02 |
| **lactate1** | af1 | 0.9693871 | 0.7219013 | 1.2995537 | 8.36E-01 | **lactate2** | af1 | 0.9541168 | 0.7688968 | 1.1833412 | 6.69E-01 | **lactate3** | af1 | 0.9061484 | 0.7340141 | 1.1181159 | 3.58E-01 |
| **lactate1** | cr1 | 1.1169587 | 0.964066 | 1.284069 | 1.38E-01 | **lactate2** | cr2 | 1.0585891 | 0.9471476 | 1.1799492 | 3.12E-01 | **lactate3** | cr3 | 1.0049088 | 0.9007983 | 1.1189904 | 9.29E-01 |
| **lactate1** | na1 | 0.9721001 | 0.8354165 | 1.1341979 | 7.17E-01 | **lactate2** | na2 | 1.0565127 | 0.9556172 | 1.1678485 | 2.83E-01 | **lactate3** | na3 | 1.1267753 | 1.0221358 | 1.2428113 | 1.64E-02 |
| **lactate1** | bicarb1 | 1.7316338 | 1.4793815 | 2.0311106 | 5.64E-12 | **lactate2** | bicarb2 | 1.5110397 | 1.3520702 | 1.6916915 | 1.48E-13 | **lactate3** | bicarb3 | 1.3491337 | 1.2117331 | 1.5041209 | 3.65E-08 |
| **lactate1** | hgb1 | 1.3507286 | 1.1569428 | 1.5788817 | 1.37E-04 | **lactate2** | hgb2 | 1.4648308 | 1.323049 | 1.6233762 | 1.31E-13 | **lactate3** | hgb3 | 1.3379403 | 1.2080633 | 1.483019 | 1.99E-08 |
| **lactate1** | wbc1 | 0.5004426 | 0.4044766 | 0.6133875 | 1.49E-12 | **lactate2** | wbc2 | 0.5630645 | 0.4889395 | 0.6455079 | 0.00E+00 | **lactate3** | wbc3 | 0.5775168 | 0.5108316 | 0.6505885 | 0.00E+00 |
| **lactate1** | plt1 | 1.4138164 | 1.2286953 | 1.6210227 | 2.18E-06 | **lactate2** | plt2 | 1.5995043 | 1.4474364 | 1.7716241 | 0.00E+00 | **lactate3** | plt3 | 1.5739901 | 1.4198142 | 1.7494599 | 0.00E+00 |
| **lactate1** | hr1 | 0.8620436 | 0.7457026 | 0.9937821 | 4.07E-02 | **lactate2** | hr2 | 0.7402111 | 0.6659837 | 0.8212198 | 8.64E-09 | **lactate3** | hr3 | 0.713747 | 0.644428 | 0.7892661 | 2.61E-11 |
| **lactate1** | sbp1 | 0.9500142 | 0.8022731 | 1.1220547 | 5.48E-01 | **lactate2** | sbp2 | 0.8181805 | 0.7309586 | 0.9149656 | 4.23E-04 | **lactate3** | sbp3 | 0.9475449 | 0.8485114 | 1.0576783 | 3.37E-01 |
| **lactate1** | dbp1 | 1.1336794 | 0.9475463 | 1.3589666 | 1.71E-01 | **lactate2** | dbp2 | 1.3127497 | 1.1683156 | 1.4767421 | 4.30E-06 | **lactate3** | dbp3 | 1.2397928 | 1.1061305 | 1.3912798 | 2.11E-04 |
| **lactate1** | **AUC = 0.816** | | | | | **lactate2** | **AUC = 0.772** | | | | | **lactate3** | **AUC = 0.764** | | | | |
| **alt1** | age | 1.0534491 | 0.8312929 | 1.3443736 | 6.70E-01 | **alt2** | age | 1.2948386 | 1.1332894 | 1.4821977 | 1.33E-04 | **alt3** | age | 1.2569102 | 1.1084689 | 1.4275801 | 3.42E-04 |
| **alt1** | sexMale | 0.9803111 | 0.6264017 | 1.5593202 | 9.32E-01 | **alt2** | sexMale | 0.9998411 | 0.7787382 | 1.2878106 | 9.99E-01 | **alt3** | sexMale | 0.8869124 | 0.7007307 | 1.1249864 | 3.21E-01 |
| **alt1** | bmi | 0.7194558 | 0.5662583 | 0.9002082 | 3.40E-03 | **alt2** | bmi | 1.0712155 | 0.9534803 | 1.200625 | 2.45E-01 | **alt3** | bmi | 1.0630449 | 0.9517343 | 1.1857302 | 2.77E-01 |
| **alt1** | csetiologyami | 0.5806587 | 0.3315143 | 0.9911402 | 4.62E-02 | **alt2** | csetiologyami | 0.9090996 | 0.699297 | 1.1795953 | 4.74E-01 | **alt3** | csetiologyami | 0.926035 | 0.7223446 | 1.1854826 | 5.43E-01 |
| **alt1** | htn1 | 0.6774521 | 0.4338334 | 1.0736037 | 9.63E-02 | **alt2** | htn1 | 0.9740502 | 0.7546723 | 1.2630789 | 8.41E-01 | **alt3** | htn1 | 1.2504873 | 0.9765045 | 1.6086394 | 7.67E-02 |
| **alt1** | ckd1 | 1.2266039 | 0.7624151 | 1.9672341 | 3.98E-01 | **alt2** | ckd1 | 1.0132018 | 0.7656472 | 1.337196 | 9.27E-01 | **alt3** | ckd1 | 0.9203499 | 0.7040936 | 1.2001054 | 5.41E-01 |
| **alt1** | hf1 | 0.6118826 | 0.3683741 | 1.0257906 | 6.22E-02 | **alt2** | hf1 | 0.7953983 | 0.6107399 | 1.0369302 | 9.05E-02 | **alt3** | hf1 | 0.6108315 | 0.4765678 | 0.7826084 | 9.78E-05 |
| **alt1** | cad1 | 1.2179959 | 0.7955728 | 1.8670568 | 3.64E-01 | **alt2** | cad1 | 1.2540879 | 0.9883314 | 1.5920913 | 6.24E-02 | **alt3** | cad1 | 1.1694444 | 0.932899 | 1.4665465 | 1.75E-01 |
| **alt1** | dm1 | 1.4892983 | 0.9719574 | 2.2894081 | 6.73E-02 | **alt2** | dm1 | 0.8541936 | 0.6712148 | 1.0853391 | 1.97E-01 | **alt3** | dm1 | 0.8432039 | 0.6721244 | 1.0566541 | 1.39E-01 |
| **alt1** | af1 | 1.1693637 | 0.7490637 | 1.8301339 | 4.91E-01 | **alt2** | af1 | 1.0307369 | 0.8028837 | 1.3223313 | 8.12E-01 | **alt3** | af1 | 0.8716954 | 0.6865487 | 1.1054823 | 2.58E-01 |
| **alt1** | cr1 | 1.1388461 | 0.9080773 | 1.3871351 | 2.46E-01 | **alt2** | cr2 | 0.8847335 | 0.7609989 | 1.0183723 | 8.94E-02 | **alt3** | cr3 | 0.9166821 | 0.8004598 | 1.0426441 | 1.89E-01 |
| **alt1** | na1 | 1.2317447 | 0.9829766 | 1.5504236 | 7.04E-02 | **alt2** | na2 | 1.1596989 | 1.0310554 | 1.3068361 | 1.33E-02 | **alt3** | na3 | 1.2660431 | 1.1323316 | 1.4172555 | 3.21E-05 |
| **alt1** | bicarb1 | 1.9857602 | 1.6102726 | 2.4553112 | 1.60E-10 | **alt2** | bicarb2 | 1.2414914 | 1.0943447 | 1.4089473 | 7.78E-04 | **alt3** | bicarb3 | 1.3093966 | 1.163773 | 1.4742809 | 7.22E-06 |
| **alt1** | hgb1 | 1.0699451 | 0.8480992 | 1.3483604 | 5.67E-01 | **alt2** | hgb2 | 1.124763 | 0.9975398 | 1.2678241 | 5.49E-02 | **alt3** | hgb3 | 1.0334896 | 0.9199811 | 1.1602929 | 5.78E-01 |
| **alt1** | wbc1 | 0.7525392 | 0.5542745 | 0.9949865 | 4.58E-02 | **alt2** | wbc2 | 0.8684846 | 0.74903 | 0.9981917 | 4.70E-02 | **alt3** | wbc3 | 0.9520643 | 0.8418568 | 1.0724961 | 4.23E-01 |
| **alt1** | plt1 | 1.3037543 | 1.0736915 | 1.5596702 | 8.32E-03 | **alt2** | plt2 | 1.2069512 | 1.0812037 | 1.3437018 | 9.45E-04 | **alt3** | plt3 | 1.2693615 | 1.1442793 | 1.408037 | 9.09E-06 |
| **alt1** | hr1 | 0.9308561 | 0.7492391 | 1.148014 | 5.08E-01 | **alt2** | hr2 | 0.9036908 | 0.8002652 | 1.0182106 | 9.66E-02 | **alt3** | hr3 | 0.7965381 | 0.7085338 | 0.8935759 | 9.25E-05 |
| **alt1** | sbp1 | 0.7202227 | 0.5486456 | 0.9420613 | 1.64E-02 | **alt2** | sbp2 | 1.0389427 | 0.9147443 | 1.1797392 | 5.56E-01 | **alt3** | sbp3 | 0.9459264 | 0.8365759 | 1.0683168 | 3.72E-01 |
| **alt1** | dbp1 | 0.99899 | 0.7425656 | 1.3474895 | 9.95E-01 | **alt2** | dbp2 | 1.037183 | 0.9070274 | 1.1867945 | 5.94E-01 | **alt3** | dbp3 | 1.1433671 | 1.0062989 | 1.3003094 | 3.97E-02 |
| **alt1** | **AUC = 0.774** | | | | | **alt2** | **AUC = 0.658** | | | | | **alt3** | **AUC = 0.687** | | | | |
| **alb1** | age | 1.0099546 | 0.7893088 | 1.3020869 | 9.38E-01 | **alb2** | age | 1.2909391 | 1.1268469 | 1.4817685 | 2.15E-04 | **alb3** | age | 1.2857886 | 1.1307723 | 1.4646831 | 1.15E-04 |
| **alb1** | sexMale | 0.9744881 | 0.6091753 | 1.5867736 | 9.16E-01 | **alb2** | sexMale | 0.9707985 | 0.7526307 | 1.2561738 | 8.21E-01 | **alb3** | sexMale | 0.8907494 | 0.7003671 | 1.1354684 | 3.49E-01 |
| **alb1** | bmi | 0.7348112 | 0.5721386 | 0.9274352 | 8.58E-03 | **alb2** | bmi | 1.0720348 | 0.9523862 | 1.2038422 | 2.47E-01 | **alb3** | bmi | 1.0715734 | 0.9571994 | 1.1977816 | 2.28E-01 |
| **alb1** | csetiologyami | 0.6380984 | 0.3540336 | 1.1190393 | 1.18E-01 | **alb2** | csetiologyami | 0.9535977 | 0.7296436 | 1.2441344 | 7.27E-01 | **alb3** | csetiologyami | 0.9737034 | 0.7564487 | 1.2517967 | 8.36E-01 |
| **alb1** | htn1 | 0.7524935 | 0.4705414 | 1.2250697 | 2.48E-01 | **alb2** | htn1 | 0.9583703 | 0.7396128 | 1.2476508 | 7.50E-01 | **alb3** | htn1 | 1.2353811 | 0.9609767 | 1.5956142 | 9.95E-02 |
| **alb1** | ckd1 | 1.2516392 | 0.755391 | 2.0649262 | 3.82E-01 | **alb2** | ckd1 | 1.0356822 | 0.777952 | 1.3750882 | 8.09E-01 | **alb3** | ckd1 | 0.8959246 | 0.6809326 | 1.1755305 | 4.29E-01 |
| **alb1** | hf1 | 0.6192233 | 0.3630875 | 1.0665877 | 8.35E-02 | **alb2** | hf1 | 0.7602549 | 0.580676 | 0.9961697 | 4.68E-02 | **alb3** | hf1 | 0.6411865 | 0.4981092 | 0.8251977 | 5.61E-04 |
| **alb1** | cad1 | 1.2441629 | 0.7937147 | 1.9523888 | 3.40E-01 | **alb2** | cad1 | 1.3141261 | 1.0309577 | 1.6762632 | 2.74E-02 | **alb3** | cad1 | 1.2070239 | 0.9588776 | 1.5201648 | 1.09E-01 |
| **alb1** | dm1 | 1.2183252 | 0.7765845 | 1.9096559 | 3.89E-01 | **alb2** | dm1 | 0.8112193 | 0.6340452 | 1.0359299 | 9.37E-02 | **alb3** | dm1 | 0.8526456 | 0.6768553 | 1.072861 | 1.74E-01 |
| **alb1** | af1 | 1.205673 | 0.7527307 | 1.9381995 | 4.37E-01 | **alb2** | af1 | 1.0355043 | 0.8023218 | 1.3355096 | 7.88E-01 | **alb3** | af1 | 0.8579377 | 0.6725925 | 1.0929383 | 2.15E-01 |
| **alb1** | cr1 | 1.0219971 | 0.7757314 | 1.2876013 | 8.66E-01 | **alb2** | cr2 | 0.8765888 | 0.7496061 | 1.0140787 | 7.74E-02 | **alb3** | cr3 | 0.8822746 | 0.7644098 | 1.0101399 | 7.03E-02 |
| **alb1** | na1 | 1.2503895 | 0.9831896 | 1.5987129 | 6.87E-02 | **alb2** | na2 | 1.1636319 | 1.0321097 | 1.3145671 | 1.31E-02 | **alb3** | na3 | 1.2833864 | 1.1450237 | 1.4403538 | 1.67E-05 |
| **alb1** | bicarb1 | 2.0065669 | 1.6108144 | 2.5061891 | 6.47E-10 | **alb2** | bicarb2 | 1.3188921 | 1.1592779 | 1.5014171 | 2.57E-05 | **alb3** | bicarb3 | 1.3161064 | 1.1671405 | 1.4851664 | 7.19E-06 |
| **alb1** | hgb1 | 1.0859719 | 0.8510701 | 1.384238 | 5.06E-01 | **alb2** | hgb2 | 1.1272072 | 0.997497 | 1.2733904 | 5.49E-02 | **alb3** | hgb3 | 1.0687053 | 0.9499781 | 1.2016806 | 2.68E-01 |
| **alb1** | wbc1 | 0.6976832 | 0.4997441 | 0.9453673 | 1.90E-02 | **alb2** | wbc2 | 0.8399132 | 0.7195348 | 0.972311 | 1.86E-02 | **alb3** | wbc3 | 0.9464024 | 0.8339358 | 1.0695595 | 3.82E-01 |
| **alb1** | plt1 | 1.2569225 | 1.0193325 | 1.5230357 | 3.31E-02 | **alb2** | plt2 | 1.2320686 | 1.1016475 | 1.3741896 | 3.19E-04 | **alb3** | plt3 | 1.2661138 | 1.1393584 | 1.4061513 | 1.62E-05 |
| **alb1** | hr1 | 1.0076017 | 0.8051024 | 1.2512124 | 9.46E-01 | **alb2** | hr2 | 0.9042758 | 0.7985505 | 1.0216008 | 1.07E-01 | **alb3** | hr3 | 0.7962153 | 0.7066152 | 0.8952107 | 1.22E-04 |
| **alb1** | sbp1 | 0.7725351 | 0.581089 | 1.0237187 | 7.25E-02 | **alb2** | sbp2 | 1.0534924 | 0.9247574 | 1.199929 | 4.33E-01 | **alb3** | sbp3 | 0.9268069 | 0.8170315 | 1.050002 | 2.33E-01 |
| **alb1** | dbp1 | 0.9139198 | 0.6702802 | 1.2497814 | 5.72E-01 | **alb2** | dbp2 | 1.0532936 | 0.9184361 | 1.2088357 | 4.58E-01 | **alb3** | dbp3 | 1.1884286 | 1.0426623 | 1.3560325 | 9.65E-03 |
| **alb1** | **AUC = 0.777** | | | | | **alb2** | **AUC = 0.673** | | | | | **alb3** | **AUC = 0.695** | | | | |

| **Table S3. Eigenvalues derived from parallel analysis on FAMD coordinates** | | | | | | |
| --- | --- | --- | --- | --- | --- | --- |
| Dimension | Day1 | | Day 2 | | Day 3 | |
|  | Observed | Simulated  (n = 500, 95^th^ percentile) | Observed | Simulated  (n = 500, 95^th^ percentile) | Observed | Simulated  (n = 500, 95^th^ percentile) |
| 1 | 2.875 | 1.188 | 2.695 | 1.195 | 2.554 | 1.206 |
| 2 | 2.620 | 1.155 | 2.385 | 1.164 | 2.275 | 1.169 |
| 3 | 1.880 | 1.134 | 1.859 | 1.141 | 1.845 | 1.146 |
| 4 | 1.576 | 1.115 | 1.462 | 1.121 | 1.483 | 1.128 |
| 5 | 1.319 | 1.098 | 1.320 | 1.104 | 1.353 | 1.110 |
| 6 | 1.170 | 1.084 | 1.200 | 1.087 | 1.218 | 1.093 |
| 7 | 1.131 | 1.070 | 1.108 | 1.073 | 1.151 | 1.077 |
| 8 | 1.058 | 1.058 | 1.054 | 1.061 | 1.029 | 1.063 |
| 9 | 0.963 | 1.044 | 0.977 | 1.046 | 1.005 | 1.048 |
| 10 | 0.875 | 1.032 | 0.928 | 1.033 | 0.933 | 1.036 |
| 11 | 0.832 | 1.020 | 0.885 | 1.021 | 0.895 | 1.021 |
| 12 | 0.771 | 1.008 | 0.816 | 1.009 | 0.821 | 1.008 |
| FAMD = Factor Analysis of Mixed Data | | | | | | |

| **Table S4. NBClust cluster indices of seven-dimensional FAMD coordinates** | | | | | | | | | | | | | | |
| --- | --- | --- | --- | --- | --- | --- | --- | --- | --- | --- | --- | --- | --- | --- |
| **Day** | **k** | **KL** | **CH** | **Hartigan** | **CCC** | **Scott** | **Marriot** | **TrCovW** | **TraceW** | **Friedman** | **Rubin** | **Cindex** | **DB** | **Silhouette** |
| **1** | 2 | 5.3028 | 315.2445 | 301.8998 | -26.815 | 1902.031 | 1.4163691528461e+26 | 26093943 | 32952.5 | 0.6656 | 1.1086 | 0.3169 | 2.9771 | 0.1073 |
|  | 3 | 1.3707 | 324.8466 | 210.5292 | -33.7948 | 3424.823 | 1.88704203522716e+26 | 19674977 | 29849.36 | 1.4338 | 1.2238 | 0.3693 | 2.2905 | 0.0963 |
|  | 4 | 1.2682 | 302.3422 | 161.7909 | -38.8057 | 4764.33 | 2.11579268873162e+26 | 16697546 | 27831.02 | 2.022 | 1.3126 | 0.3578 | 2.4821 | 0.0927 |
|  | 5 | 0.9013 | 279.7499 | 123.3895 | -43.2118 | 5752.758 | 2.35274550556098e+26 | 14784290 | 26361.33 | 2.4747 | 1.3857 | 0.3512 | 2.0786 | 0.0894 |
|  | 6 | 6.8772 | 257.9079 | 126.0103 | -47.3515 | 6574.905 | 2.55311026131433e+26 | 13553518 | 25285.84 | 2.918 | 1.4447 | 0.3517 | 2.384 | 0.0693 |
|  | 7 | 0.1934 | 245.1792 | 89.44 | -49.3119 | 7370.347 | 2.64293429090525e+26 | 12334801 | 24232.88 | 3.355 | 1.5074 | 0.3442 | 2.3248 | 0.0699 |
|  | 8 | 2.1997 | 229.3353 | 88.6799 | -52.9723 | 8057.25 | 2.72529993661807e+26 | 11602904 | 23507.62 | 3.7311 | 1.5539 | 0.3387 | 2.327 | 0.0614 |
|  | **k** | **Duda** | **Pseudot2** | **Beale** | **Ratkowsky** | **Ball** | **Ptbiserial** | **Frey** | **McClain** | **Dunn** | **Hubert** | **SDindex** | **Dindex** | **SDbw** |
|  | 2 | 0.7757 | 323.6606 | 1.3195 | 0.1375 | 16476.25 | 0.2206 | -0.253 | 0.7953 | 0.059 | 1e-04 | 1.6414 | 3.2398 | 2.0989 |
|  | 3 | 0.8944 | 210.4201 | 0.5385 | 0.1595 | 9949.787 | 0.2815 | 0.2559 | 1.0179 | 0.0707 | 1e-04 | 1.3049 | 3.0943 | 3.7854 |
|  | 4 | 0.7644 | 170.489 | 1.4051 | 0.184 | 6957.755 | 0.3227 | -0.0373 | 1.9501 | 0.0732 | 1e-04 | 1.3253 | 2.9909 | 1.4357 |
|  | 5 | 0.901 | 134.901 | 0.5012 | 0.1879 | 5272.267 | 0.34 | 0.9389 | 2.0896 | 0.0732 | 1e-04 | 1.1334 | 2.9084 | 0.8116 |
|  | 6 | 0.7921 | 113.9126 | 1.1957 | 0.1847 | 4214.307 | 0.2981 | -0.0208 | 3.4484 | 0.0753 | 1e-04 | 1.3272 | 2.8412 | 0.75 |
|  | 7 | 0.872 | 100.3016 | 0.6695 | 0.1817 | 3461.84 | 0.312 | 0.3706 | 3.6495 | 0.0753 | 1e-04 | 1.3737 | 2.7857 | 0.6597 |
|  | 8 | 0.8708 | 96.5634 | 0.6762 | 0.1783 | 2938.453 | 0.3041 | 0.3479 | 4.2709 | 0.0753 | 1e-04 | 1.3625 | 2.7411 | 0.6358 |
| **2** | **k** | **KL** | **CH** | **Hartigan** | **CCC** | **Scott** | **Marriot** | **TrCovW** | **TraceW** | **Friedman** | **Rubin** | **Cindex** | **DB** | **Silhouette** |
|  | 2 | 14.525 | 284.4951 | 303.2391 | -24.7693 | 1788.986 | 5.23409574771909e+25 | 17494487 | 28414.45 | 0.6952 | 1.1087 | 0.315 | 2.4891 | 0.0859 |
|  | 3 | 0.2134 | 310.2311 | 144.0224 | -27.7232 | 3355.687 | 6.47480313454482e+25 | 13825860 | 25463.88 | 1.6862 | 1.2372 | 0.3203 | 2.3473 | 0.1041 |
|  | 4 | 2.5655 | 266.1128 | 141.8494 | -36.5958 | 4439.465 | 7.6100422852698e+25 | 12402161 | 24135.13 | 2.1957 | 1.3053 | 0.309 | 2.5718 | 0.0791 |
|  | 5 | 0.7337 | 245.7793 | 99.5677 | -40.5703 | 5266.421 | 8.67116570232887e+25 | 10923270 | 22893.3 | 2.7007 | 1.3761 | 0.2997 | 2.4609 | 0.0801 |
|  | 6 | 1.067 | 223.9407 | 80.794 | -45.6346 | 5968.158 | 9.55157020552439e+25 | 10041083 | 22053.28 | 3.0999 | 1.4285 | 0.2953 | 2.4028 | 0.0554 |
|  | 7 | 1.8518 | 205.7744 | 81.9394 | -50.518 | 6495.426 | 1.06300296656887e+26 | 9339554 | 21391.85 | 3.432 | 1.4727 | 0.2913 | 2.3953 | 0.0455 |
|  | 8 | 2.5628 | 193.5426 | 81.7571 | -54.0065 | 7017.645 | 1.13742191119725e+26 | 8700852 | 20741.19 | 3.7237 | 1.5189 | 0.2862 | 2.4071 | 0.0428 |
|  | **k** | **Duda** | **Pseudot2** | **Beale** | **Ratkowsky** | **Ball** | **Ptbiserial** | **Frey** | **McClain** | **Dunn** | **Hubert** | **SDindex** | **Dindex** | **SDbw** |
|  | 2 | 0.8739 | 281.3806 | 0.6585 | 0.144 | 14207.23 | 0.1089 | -0.4007 | 0.5733 | 0.0679 | 0 | 1.5138 | 3.1455 | 1.3424 |
|  | 3 | 0.9019 | 156.7025 | 0.4965 | 0.1551 | 8487.96 | 0.3079 | 0.7949 | 1.2124 | 0.0686 | 1e-04 | 1.3888 | 2.9961 | 1.4262 |
|  | 4 | 0.8203 | 111.3234 | 0.9986 | 0.1812 | 6033.784 | 0.2881 | -0.15 | 2.1866 | 0.0686 | 1e-04 | 1.458 | 2.911 | 1.3668 |
|  | 5 | 0.8972 | 111.0185 | 0.5226 | 0.1821 | 4578.66 | 0.322 | 0.9355 | 2.3214 | 0.0686 | 1e-04 | 1.6227 | 2.8462 | 0.8916 |
|  | 6 | 0.8917 | 89.7145 | 0.5536 | 0.181 | 3675.547 | 0.2994 | 0.7408 | 3.0659 | 0.0686 | 1e-04 | 1.5405 | 2.7835 | 0.7285 |
|  | 7 | 0.8703 | 99.117 | 0.6795 | 0.1753 | 3055.978 | 0.2812 | 0.3346 | 3.8701 | 0.0686 | 1e-04 | 1.5473 | 2.7372 | 0.6909 |
|  | 8 | 0.8441 | 86.6445 | 0.8417 | 0.1715 | 2592.649 | 0.2733 | 0.1411 | 4.648 | 0.0686 | 1e-04 | 1.4865 | 2.6936 | 0.6651 |
| **3** | **k** | **KL** | **CH** | **Hartigan** | **CCC** | **Scott** | **Marriot** | **TrCovW** | **TraceW** | **Friedman** | **Rubin** | **Cindex** | **DB** | **Silhouette** |
|  | 2 | 3.921 | 250.7048 | 230.9743 | -23.4414 | 1442.878 | 2.95517289060592e+25 | 14163449 | 25714.78 | 0.7317 | 1.1049 | 0.3447 | 2.5833 | 0.1437 |
|  | 3 | 1.3803 | 252.848 | 161.5704 | -29.1867 | 2863.125 | 3.67200783845033e+25 | 11955564 | 23448.66 | 1.4821 | 1.2117 | 0.3268 | 2.6002 | 0.0853 |
|  | 4 | 0.9629 | 233.7245 | 114.0385 | -34.9106 | 3806.678 | 4.40015886034026e+25 | 10051268 | 21963.26 | 2.0564 | 1.2936 | 0.3138 | 2.4624 | 0.0774 |
|  | 5 | 3.1329 | 212.0853 | 114.0989 | -40.1107 | 4678.181 | 4.7759231377112e+25 | 9490913 | 20962.21 | 2.509 | 1.3554 | 0.3101 | 2.3775 | 0.072 |
|  | 6 | 3.0244 | 200.5141 | 103.004 | -43.1412 | 5435.001 | 5.01199463540824e+25 | 8748327 | 20005.93 | 3.0013 | 1.4202 | 0.3041 | 2.3646 | 0.0566 |
|  | 7 | 0.5853 | 191.3957 | 85.2498 | -45.291 | 6196.85 | 4.9611443868198e+25 | 8112645 | 19178.01 | 3.4214 | 1.4815 | 0.2971 | 2.3115 | 0.0602 |
|  | 8 | 7.4324 | 182.0196 | 82.5969 | -47.8216 | 6728.511 | 5.18844060894683e+25 | 7550310 | 18516.17 | 3.7306 | 1.5345 | 0.2919 | 2.2653 | 0.0579 |
|  | **k** | **Duda** | **Pseudot2** | **Beale** | **Ratkowsky** | **Ball** | **Ptbiserial** | **Frey** | **McClain** | **Dunn** | **Hubert** | **SDindex** | **Dindex** | **SDbw** |
|  | 2 | 0.8831 | 250.779 | 0.6039 | 0.1089 | 12857.39 | 0.2908 | 1.4486 | 0.4093 | 0.078 | 1e-04 | 1.4735 | 3.1724 | 1.5615 |
|  | 3 | 0.851 | 174.73 | 0.7986 | 0.156 | 7816.219 | 0.2644 | 0.2302 | 1.5178 | 0.0665 | 1e-04 | 1.3816 | 3.0109 | 2.1802 |
|  | 4 | 0.8417 | 92.701 | 0.8568 | 0.1677 | 5490.815 | 0.2826 | -0.0836 | 2.211 | 0.0665 | 1e-04 | 1.303 | 2.909 | 1.6177 |
|  | 5 | 0.8663 | 138.087 | 0.7037 | 0.1753 | 4192.443 | 0.3076 | 1.0134 | 2.3936 | 0.0674 | 1e-04 | 1.2806 | 2.8461 | 0.7597 |
|  | 6 | 0.8113 | 108.355 | 1.0594 | 0.1835 | 3334.321 | 0.2727 | 0.0794 | 3.6225 | 0.0674 | 1e-04 | 1.3007 | 2.7719 | 0.8461 |
|  | 7 | 0.8384 | 102.1565 | 0.8784 | 0.1879 | 2739.716 | 0.2809 | 0.2891 | 4.057 | 0.0674 | 1e-04 | 1.2634 | 2.7131 | 0.7248 |
|  | 8 | 0.8384 | 58.9867 | 0.8773 | 0.1879 | 2314.521 | 0.2751 | -0.0687 | 4.768 | 0.0674 | 1e-04 | 1.2155 | 2.6624 | 0.6449 |

| **Table S5. Sensitivity analysis of clustering characteristics using only complete cases** | | | | | | | | | | | | |
| --- | --- | --- | --- | --- | --- | --- | --- | --- | --- | --- | --- | --- |
|  | **Day 1**  **(n = 2300)** | | | | **Day 2**  **(n = 1574)** | | | | **Day 3**  **(n = 1225)** | | | |
| **Variables** | **Cardiac_1_  (n = 887)** | **CR_1_  (n = 896)** | **CM_1_**  **(n = 517)** | **P value** | **Cardiac_2_  (n = 657)** | **CR_2_  (n = 589)** | **CM_2_**  **(n = 328)** | **P value** | **Cardiac_3_  (n = 516)** | **CR_3_  (n = 496)** | **CM_3_**  **(n = 213)** | **P value** |
| **Age (year)** | 63.94 (13.86) | 72.56 (11.23) | 69.49 (13.67) | <0.001 | 64.11 (13.72) | 69.44 (12.40) | 69.81 (12.43) | <0.001 | 65.13 (13.17) | 67.45 (12.63) | 68.89 (12.91) | <0.001 |
| **Male (%)** | 620 (69.90%) | 588 (65.62%) | 303 (58.61%) | <0.001 | 448 (68.19%) | 407 (69.10%) | 222 (67.68%) | 0.894 | 371 (71.90%) | 368 (74.19%) | 143 (67.14%) | 0.158 |
| **BMI (kg/m2)** | 27.87 (6.22) | 28.01 (7.03) | 27.51 (6.22) | 0.377 | 28.25 (5.90) | 27.31 (6.68) | 27.41 (6.36) | 0.020 | 28.14 (5.89) | 27.28 (6.68) | 27.01 (5.46) | 0.027 |
| **BSA (m2)** | 1.95 (0.28) | 1.93 (0.29) | 1.90 (0.29) | 0.014 | 1.95 (0.28) | 1.93 (0.30) | 1.92 (0.29) | 0.193 | 1.95 (0.28) | 1.95 (0.30) | 1.91 (0.25) | 0.105 |
| **AMI-CS (%)** | 504 (56.82%) | 102 (11.38%) | 265 (51.26%) | <0.001 | 389 (59.21%) | 68 (11.54%) | 161 (49.09%) | <0.001 | 338 (65.50%) | 41 (8.27%) | 104 (48.83%) | <0.001 |
| **Sodium (mEq/L)** | 136.66 (4.51) | 134.97 (5.51) | 135.20 (6.51) | <0.001 | 137.60 (4.39) | 135.25 (4.92) | 136.55 (5.36) | <0.001 | 137.45 (4.67) | 135.11 (4.83) | 135.64 (5.06) | <0.001 |
| **ALT (IU/L)** | 117.59 (305.90) | 110.12 (299.99) | 1042.04 (1602.04) | <0.001 | 118.15 (345.54) | 186.29 (455.56) | 1169.12 (1674.79) | <0.001 | 114.67 (359.98) | 189.91 (434.20) | 1186.37 (1583.21) | <0.001 |
| **Creatinine (mg/dL)** | 1.34 (0.62) | 2.61 (1.75) | 2.80 (1.73) | <0.001 | 1.40 (0.87) | 2.29 (1.32) | 3.09 (1.63) | <0.001 | 1.53 (1.06) | 2.14 (1.34) | 2.92 (1.64) | <0.001 |
| **Hemoglobin (g/dL)** | 11.47 (2.31) | 10.36 (2.04) | 9.87 (2.32) | <0.001 | 10.35 (2.08) | 10.49 (2.08) | 9.63 (1.89) | <0.001 | 9.81 (1.77) | 10.71 (2.16) | 9.35 (1.81) | <0.001 |
| **Platelet (k/uL)** | 192.66 (82.87) | 173.88 (75.74) | 155.66 (88.69) | <0.001 | 153.57 (73.61) | 170.41 (80.39) | 120.68 (77.79) | <0.001 | 140.06 (65.09) | 167.63 (86.73) | 98.51 (63.34) | <0.001 |
| **WBC (k/uL)** | 16.39 (6.86) | 11.23 (5.35) | 20.07 (8.45) | <0.001 | 14.60 (5.52) | 10.25 (4.41) | 17.41 (9.80) | <0.001 | 13.99 (4.90) | 9.87 (4.36) | 15.90 (6.70) | <0.001 |
| **Bicarbonate (mEq/L)** | 19.75 (3.47) | 21.19 (4.51) | 14.09 (4.04) | <0.001 | 21.70 (3.13) | 23.15 (4.28) | 17.44 (4.14) | <0.001 | 21.96 (3.44) | 23.74 (4.44) | 18.50 (4.35) | <0.001 |
| **Lactate (mmol/L)** | 3.57 (2.56) | 2.84 (2.17) | 9.96 (5.09) | <0.001 | 2.02 (1.30) | 1.69 (0.93) | 6.05 (4.79) | <0.001 | 1.65 (0.88) | 1.57 (0.74) | 4.94 (4.18) | <0.001 |
| **HR (BPM)** | 90.51 (18.76) | 91.22 (20.87) | 94.56 (24.23) | 0.002 | 89.52 (17.91) | 95.40 (19.34) | 98.24 (22.47) | <0.001 | 89.54 (16.26) | 97.66 (19.27) | 99.80 (23.22) | <0.001 |
| **SBP (mmHg)** | 97.99 (19.70) | 87.32 (19.48) | 80.00 (23.01) | <0.001 | 100.51 (19.45) | 97.48 (18.27) | 88.56 (21.05) | <0.001 | 102.23 (21.05) | 98.66 (16.87) | 91.77 (20.62) | <0.001 |
| **DBP (mmHg)** | 54.03 (13.94) | 47.36 (12.37) | 42.46 (15.34) | <0.001 | 50.71 (11.81) | 51.44 (11.95) | 47.19 (13.96) | <0.001 | 50.31 (12.59) | 54.08 (11.77) | 47.61 (13.47) | <0.001 |
| **HTN (%)** | 526 (59.30%) | 733 (81.81%) | 360 (69.63%) | <0.001 | 394 (59.97%) | 457 (77.59%) | 238 (72.56%) | <0.001 | 324 (62.79%) | 368 (74.19%) | 146 (68.54%) | <0.001 |
| **CKD (%)** | 55 (6.20%) | 533 (59.49%) | 137 (26.50%) | <0.001 | 37 (5.63%) | 373 (63.33%) | 102 (31.10%) | <0.001 | 46 (8.91%) | 317 (63.91%) | 62 (29.11%) | <0.001 |
| **HF (%)** | 428 (48.25%) | 822 (91.74%) | 262 (50.68%) | <0.001 | 277 (42.16%) | 569 (96.60%) | 199 (60.67%) | <0.001 | 235 (45.54%) | 481 (96.98%) | 134 (62.91%) | <0.001 |
| **CAD (%)** | 319 (35.96%) | 527 (58.82%) | 205 (39.65%) | <0.001 | 240 (36.53%) | 340 (57.72%) | 144 (43.90%) | <0.001 | 247 (47.87%) | 265 (53.43%) | 91 (42.72%) | 0.024 |
| **DM (%)** | 268 (30.21%) | 518 (57.81%) | 227 (43.91%) | <0.001 | 208 (31.66%) | 358 (60.78%) | 162 (49.39%) | <0.001 | 206 (39.92%) | 282 (56.85%) | 102 (47.89%) | <0.001 |
| **AF (%)** | 194 (21.87%) | 595 (66.41%) | 156 (30.17%) | <0.001 | 152 (23.14%) | 381 (64.69%) | 116 (35.37%) | <0.001 | 141 (27.33%) | 298 (60.08%) | 91 (42.72%) | <0.001 |
| **Death (%)** | 136 (15.33%) | 244 (27.23%) | 287 (55.51%) | <0.001 | 105 (15.98%) | 130 (22.07%) | 191 (58.23%) | <0.001 | 107 (20.74%) | 110 (22.18%) | 127 (59.62%) | <0.001 |
| AF = atrial fibrillation; ALT = alanine transaminase; AMI-CS = acute myocardial infarction-related cardiogenic shock; BPM = beats per minute; BSA = body surface area; CAD = coronary artery disease; CKD = chronic kidney disease; DBP = diastolic blood pressure; DM = diabetes mellitus; HF = heart failure; HR = heart rate; HTN = hypertension; SBP = systolic blood pressure; STEMI = ST-segment elevation myocardial infarction; WBC = white blood cell | | | | | | | | | | | | |

| **Table S6. Overall cohort characteristic (n=2906)** | | | |
| --- | --- | --- | --- |
| Parameter | Value | Parameter | Value |
| **Baseline characteristics** | | **Hemodynamics** | |
| Age (year) | 70 (60, 79) | HR (BPM) | 89 (76, 104) |
| BMI (kg/m^2^) | 26.7 (23.5, 31.0) | SBP (mmHg) | 90 (76, 103) |
| Male (%) | 1920 (66) | DBP (mmHg) | 49 (40, 57) |
| AMI-CS (%) | 1171 (40) | CO (L/min) | 4.1 (3.2, 5.2) |
| nonAMI-CS (%) | 1735 (60) | CI (L/min/m^2^) | 2.1 (1.7, 2.6) |
| **Treating centers MCS capability (%)** | | CVP (mmHg) | 11 (7, 15) |
| Durable LVAD capable | 1343 (46) | PAS (mmHg) | 38 (31, 49) |
| ECMO capable | 1317 (45) | PAD (mmHg) | 18 (14, 24) |
| pVAD capable | 97 (3) | **Laboratory** | |
| No PCI capability | 149 (5) | Sodium (mEq/L) | 136 (133, 139) |
| **Comorbidities (%)** | | Albumin (g/dL) | 3.2 (2.8, 3.5) |
| HTN | 2020 (70) | ALT (IU/L) | 45 (22, 149) |
| CKD | 915 (31) | Bilirubin (mg/dL) | 1.0 (0.6, 1.6) |
| CHF | 1862 (64) | eGFR (mL/min/1.73m2) | 41 (23, 66) |
| CAD | 1309 (45) | Hemoglobin (g/dL) | 10.7 (9, 12.5) |
| DM | 1278 (44) | Platelet (k/uL) | 172 (124, 225) |
| AF | 1179 (41) | WBC (k/uL) | 13.1 (9.3, 18.4) |
| **Hospital outcome** | | Bicarbonate (mEq/L) | 20 (17, 22) |
| Death (%) | 845 (29) | Lactate (mmol/L) | 2.9 (1.7, 5.7) |
| AF = atrial fibrillation; ALT = alanine transaminase; AMI-CS = acute myocardial infarction-related cardiogenic shock; BPM = beats per minute; BSA = body surface area; CAD = coronary artery disease; CCI = Charlson comorbidity index; CI = cardiac index; CKD = chronic kidney disease; CO = cardiac output; CVP = central venous pressure; DBP = diastolic blood pressure; DM = diabetes mellitus; ECMO = extracorporeal membrane oxygenation; eGFR = estimated glomerular filtration rate; HF = heart failure; HR = heart rate; HTN = hypertension; INR = international normalized ratio; LVAD = left ventricular assist device; PAD = pulmonary artery diastolic pressure; PAS = pulmonary artery systolic pressure; PCI = percutaneous coronary intervention; pVAD = percutaneous ventricular assist device; SBP = systolic blood pressure; STEMI = ST-segment elevation myocardial infarction; WBC = white blood cell | | | |

| **Table S7. Characteristics of day 2 and 3 phenotypes** | | | | | | | | |
| --- | --- | --- | --- | --- | --- | --- | --- | --- |
|  | **Day 2**  **(n = 2619)** | | | | **Day 3**  **(n = 2392)** | | | |
| **Variables** | **Cardiac_2_  (n = 1041)** | **CR_2_  (n = 1113)** | **CM_2_**  **(n = 465)** | **P value** | **Cardiac  (n = 984)** | **CR  (n = 1005)** | **CM**  **(n = 403)** | **P value** |
| **Age (year)** | 64 [55, 73] | 73 [64, 80] | 71 [62, 79] | <0.001 | 65 [56, 73] | 72 [63, 80] | 71 [62, 79] | <0.001 |
| **BMI (kg/m2)** | 26.7 [23.8, 31.1] | 26.9 [23.5, 31.4] | 26.5 [23.2, 30.8] | 0.056 | 27.1 [23.9, 31.2] | 26.9 [23.5, 31.4] | 26.2 [23.3, 30.7] | 0.101 |
| **BSA (m2)** | 1.93 [1.76, 2.10] | 1.91 [1.74, 2.11] | 1.89 [1.73, 2.06] | 0.067 | 1.93 [1.75, 2.09] | 1.92 [1.74, 2.11] | 1.90 [1.75, 2.09] | 0.457 |
| **Male (%)** | 710 (68.20%) | 729 (65.50%) | 311 (66.88%) | 0.411 | 670 (68.09%) | 674 (67.06%) | 275 (68.24%) | 0.858 |
| **AMI-CS (%)** | 642 (61.67%) | 158 (14.20%) | 219 (47.10%) | <0.001 | 621 (63.11%) | 119 (11.84%) | 185 (45.91%) | <0.001 |
| **Sodium (mEq/L)** | 138 [135, 140] | 136 [133, 139] | 136 [133, 140] | <0.001 | 137 [135, 140] | 136 [133, 139] | 136 [133, 139] | <0.001 |
| **ALT (IU/L)** | 40 [22, 80] | 28 [16, 71] | 343 [78, 1499] | <0.001 | 36.00 [21, 67] | 29 [17, 70] | 334 [66, 1343] | <0.001 |
| **Creatinine (mg/dL)** | 1.06 [0.85, 1.40] | 1.85 [1.27, 2.75] | 2.68 [1.75, 3.96] | <0.001 | 1.06 [0.84, 1.42] | 1.68 [1.20, 2.60] | 2.40 [1.54, 3.68] | <0.001 |
| **eGFR (mL/min/1.73m2)** | 72 [49, 94] | 35 [20, 55] | 22 [13, 37] | <0.001 | 72 [49, 94] | 39 [23, 60] | 26 [15, 46] | <0.001 |
| **Hemoglobin (g/dL)** | 11.1 [9.4, 12.8] | 10.1 [8.9, 11.8] | 9.4 [8.3, 10.7] | <0.001 | 10.3 [9.0, 12.1] | 10.1 [9.0, 11.9] | 9.2 [8.2, 10.4] | <0.001 |
| **Platelet (k/uL)** | 162 [117, 211] | 162 [121, 214] | 106 [69, 158] | <0.001 | 147 [107, 198] | 158 [117, 213] | 96 [59, 142] | <0.001 |
| **WBC (k/uL)** | 12.5 [9.7, 16.3] | 9.4 [7.2, 12.4] | 15.3 [11.5, 20.7] | <0.001 | 11.8 [9.2, 14.6] | 8.9 [6.9, 11.4] | 14.8 [11.0, 19.1] | <0.001 |
| **Bicarbonate (mEq/L)** | 22 [20, 24] | 23 [21, 26] | 18 [15, 21] | <0.001 | 23 [21, 25] | 24 [21, 27] | 19 [16, 22] | <0.001 |
| **Lactate (mmol/L)** | 1.5 [1.2, 2.1] | 1.4 [1.1, 2.0] | 4.1 [2.6, 7.6] | <0.001 | 1.3 [1.0, 1.8] | 1.4 [1.0, 1.8] | 2.8 [2.0, 5.4] | <0.001 |
| **HR (BPM)** | 88 [78, 100] | 90 [78, 106] | 94 [80, 114] | <0.001 | 87 [78, 100] | 92 [79, 105] | 94 [81, 112] | <0.001 |
| **SBP (mmHg)** | 98 [88, 111] | 95 [85, 108] | 88 [76, 101] | <0.001 | 100 [90, 112] | 96 [86, 108] | 93 [82, 106] | <0.001 |
| **DBP (mmHg)** | 52 [46, 59] | 51 [43, 57] | 46 [37, 56] | <0.001 | 54 [47, 61] | 52 [45, 59] | 47 [40, 56] | <0.001 |
| **HTN (%)** | 576 (55.33%) | 918 (82.48%) | 339 (72.90%) | <0.001 | 577 (58.64%) | 816 (81.19%) | 282 (69.98%) | <0.001 |
| **CKD (%)** | 47 (4.51%) | 638 (57.32%) | 155 (33.33%) | <0.001 | 56 (5.69%) | 599 (59.60%) | 129 (32.01%) | <0.001 |
| **HF (%)** | 414 (39.77%) | 1015 (91.19%) | 283 (60.86%) | <0.001 | 406 (41.26%) | 945 (94.03%) | 249 (61.79%) | <0.001 |
| **CAD (%)** | 335 (32.18%) | 654 (58.76%) | 219 (47.10%) | <0.001 | 351 (35.67%) | 588 (58.51%) | 185 (45.91%) | <0.001 |
| **DM (%)** | 292 (28.05%) | 658 (59.12%) | 225 (48.39%) | <0.001 | 321 (32.62%) | 589 (58.61%) | 180 (44.67%) | <0.001 |
| **AF (%)** | 193 (18.54%) | 724 (65.05%) | 168 (36.13%) | <0.001 | 173 (17.58%) | 657 (65.37%) | 181 (44.91%) | <0.001 |
| **Death (%)** | 132 (12.68%) | 243 (21.83%) | 264 (56.77%) | <0.001 | 131 (13.31%) | 207 (20.60%) | 204 (50.62%) | <0.001 |
| AF = atrial fibrillation; ALT = alanine transaminase; AMI-CS = acute myocardial infarction-related cardiogenic shock; BPM = beats per minute; BSA = body surface area; CAD = coronary artery disease; CKD = chronic kidney disease; DBP = diastolic blood pressure; DM = diabetes mellitus; eGFR = estimated glomerular filtration rate; HF = heart failure; HR = heart rate; HTN = hypertension; SBP = systolic blood pressure; STEMI = ST-segment elevation myocardial infarction; WBC = white blood cell | | | | | | | | |

**Figure S1. Patterns of missing data across the first three ICU day.** UpSet plots of missing-value patterns for the variables entered into the imputation models on day 1 (Panel A), day 2 (Panel B), and day 3 (Panel C).

**Figure S2. Random forest imputation scheme.** Three sequential imputations were performed, using the imputed data from prior days as predictors.

**Figure S3. Imputation density plot.** Density estimates comparing observed values (gray) with values generated by missingRanger using random forest prediction alone (PMM = 0, red) and with predictive mean matching to 5 donors (PMM =5, blue), shown for each variable on day 1 (Panel A), day 2 (Panel B), and day 3 (Panel C). PMM = 5 imputation model was used for final data generation.

**Figure S4. Correlation matrix of post-imputation variables.** Body surface area is highly correlated with body mass index (|r| = 0.74) and was excluded from the FAMD and clustering analysis to reduce collinearity.

**Figure S5. Determination of the Number of Retained Dimensions in the FAMD.** Parallel analysis for days 1, 2, and 3 (Panel A1, B1, C1), plotting observed eigenvalues (blue) against the 95th percentile of eigenvalues from 500 permutations of the data matrix (red). Corresponding scree plots (Panel A2, B2, C2) of the percentage of total inertia explained by each of the 23 dimensions. FAMD = Factor Analysis of Mixed Data.

**FIGURE S6. Reproducibility of Cluster Partitions Across Retained Dimensionalities.** Heatmaps of the pairwise adjusted Rand index (ARI) between clustering solutions obtained at every pair of retained dimensionalities from 2 to 23, shown for day 1 (top row), day 2 (middle row), and day 3 (bottom row) at k = 2, 3, and 4 clusters. On all three days, k = 3 produced uniformly high agreement, whereas k = 2 alternated between two mutually inconsistent bisections and k = 4 yielded fragmented, intermediate agreement.

**Figure S7. Cluster visualization.** 3-dimensional cluster visualization generated by the first three dimensions of the retained seven-dimensional FAMD coordinates. FAMD = Factor Analysis of Mixed Data.

**Figure S8.** **Random Forest Classifier and SHAP analysis.** Most important feature determining Cardiac cluster candidacy include a lower serum creatinine, lack of comorbidities, and AMI-CS. For Cario-Renal cluster, these are presence of comorbidities, non-AMI-CS, and lower WBC. For Cardio-Metabolic cluster, these are higher lactate, ALT, and lower bicarbonate. SHAP = SHapley Additive exPlanations.

**Figure S9. Invasive therapies utilization among day 2 and day 3 phenotypes.** Cardiac phenotypes consistently showed higher IABP use, whereas CM phenotype showed higher advanced MCS (Impella and VA-ECMO) use, along with the highest rate of mechanical ventilation and renal replacement therapy. CR phenotype, on the other hand, showed the lowest rate of MCS use. CM = Cardio-Metabolic; CR = Cardio-Renal; IABP = intra-aortic balloon pump; MCS = mechanical circulatory support; VA-ECMO = Veno-arterial extracorporeal membrane oxygenation.

**Figure S10. Dendrograms.** Dendrograms were generated by hierarchical clustering algorithms for visual representations.
